# Dual-isoform hUBE3A gene transfer improves behavioral and seizure outcomes in Angelman syndrome model mice

**DOI:** 10.1172/jci.insight.144712

**Published:** 2021-10-22

**Authors:** Matthew C. Judson, Charles Shyng, Jeremy M. Simon, Courtney R. Davis, A. Mattijs Punt, Mirabel T. Salmon, Noah W. Miller, Kimberly D. Ritola, Ype Elgersma, David G. Amaral, Steven J. Gray, Benjamin D. Philpot

**Affiliations:** 1 Neuroscience Center,; 2 Department of Cell Biology and Physiology,; 3 Carolina Institute for Developmental Disabilities,; 4 Gene Therapy Center, and; 5 Department of Genetics, University of North Carolina (UNC), Chapel Hill, North Carolina, USA.; 6 Department of Clinical Genetics and; 7 Department of Neuroscience, Erasmus MC University Medical Center, Rotterdam, The Netherlands.; 8 Biology and Information Science Programs and; 9 Department of Pharmacology, UNC, Chapel Hill, North Carolina, USA.; 10 Scientific Operations Manager-Viral Tools, Janelia Research Campus, Howard Hughes Medical Institute, Ashburn, Virginia, USA.; 11 ENCORE Expertise Center for Neurodevelopmental Disorders, Erasmus MC University Medical Center, Rotterdam, The Netherlands.; 12 Department of Psychiatry and Behavioral Sciences, MIND Institute, and; 13 California National Primate Research Center, University of California, Davis, California, USA.; 14 Department of Pediatrics and; 15 Eugene McDermott Center for Human Growth and Development, University of Texas (UT) Southwestern Medical Center, Dallas, Texas, USA.

**Keywords:** Neuroscience, Therapeutics, Gene therapy, Neurodevelopment, Neurological disorders

## Abstract

Loss of the maternal *UBE3A* allele causes Angelman syndrome (AS), a debilitating neurodevelopmental disorder. Here, we devised an AS treatment strategy based on reinstating dual-isoform expression of human UBE3A (hUBE3A) in the developing brain. Kozak sequence engineering of our codon-optimized vector (hUBE3Aopt) enabled translation of both short and long hUBE3A protein isoforms at a near-endogenous 3:1 (short/long) ratio, a feature that could help to support optimal therapeutic outcomes. To model widespread brain delivery and early postnatal onset of hUBE3A expression, we packaged the hUBE3Aopt vector into PHP.B capsids and performed intracerebroventricular injections in neonates. This treatment significantly improved motor learning and innate behaviors in AS mice, and it rendered them resilient to epileptogenesis and associated hippocampal neuropathologies induced by seizure kindling. hUBE3A overexpression occurred frequently in the hippocampus but was uncommon in the neocortex and other major brain structures; furthermore, it did not correlate with behavioral performance. Our results demonstrate the feasibility, tolerability, and therapeutic potential for dual-isoform hUBE3A gene transfer in the treatment of AS.

## Graphical abstract

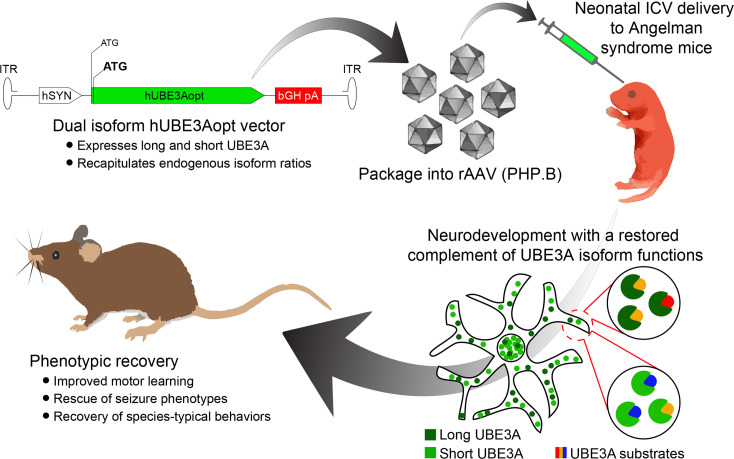

## Introduction

Angelman syndrome (AS) is a neurodevelopmental disorder characterized by severe developmental delay, motor dysfunction, absence of speech, and highly penetrant epilepsy (1, 2). While symptoms such as epilepsy can be managed, albeit with considerable difficulty (3), specific treatments for AS are lacking. AS affects ~1:20,000 individuals with deletions or mutations of the maternally inherited *UBE3A* allele (1). *UBE3A* encodes a HECT E3 ubiquitin ligase that regulates protein homeostasis — specifically, through the ubiquitination of protein substrates (4) and, generally, by inhibiting proteasome function via interactions with proteasomal proteins such as PSMD4 (5, 6), a non-ATPase subunit of the 19S regulatory particle. UBE3A is also implicated in the transcriptional coactivation of steroid hormone receptors (7) and possibly other genes (8, 9). Due to cell type–specific mechanisms that silence paternal *UBE3A* expression (10), mature neurons express UBE3A only from the maternal allele and, thus, are especially vulnerable to the genetic mutations that define AS. Rendered devoid of critical UBE3A functions, both neurons derived from patients with AS and neurons in AS model mice exhibit severe deficits in morphology, electrophysiology, and synaptic function and plasticity (11, 12). These deficits are compounded over the course of neurodevelopment, across neural circuits, to yield the neurological phenotypes that typify AS.

Neuron-specific UBE3A deletion is sufficient to induce AS-like phenotypes in mice, indicating that this insult is integral to AS etiology (13). The corollary, that UBE3A reinstatement in neurons can prevent or mitigate AS symptomology, is supported by recent work in AS model mice (14) and neurons derived from patients with AS alike (11), and it has spurred intense interest in the development of AS therapeutics based on this premise. A leading approach to UBE3A reinstatement centers on unsilencing paternal *UBE3A* expression in neurons. This can be achieved through small molecules (15), antisense oligonucleotides (ASOs) (16), or other agents (17–19) that block the expression of, or otherwise interfere with, the UBE3A antisense transcript (*UBE3A-ATS*). Processed from a long noncoding RNA that initiates from *SNRPN* promoters on the paternally inherited chromosome, *UBE3A-ATS* silences paternal *UBE3A* in *cis* in neurons (20). Paternal *UBE3A* unsilencing offers an attractive opportunity for UBE3A reinstatement under control of an endogenous promoter, presumably recapitulating optimal patterns and levels of UBE3A expression, including the proper representation of protein isoforms. Whether this advantage of unsilencing drugs outweighs their potential pitfalls — toxicity, off-target effects, and, excepting CRISPR-based gene therapies (18, 19), the need for repeated dosing — will be determined through clinical trials, some of which are currently underway (NCT04259281, NCT04428281).

Adeno-associated virus–mediated (AAV-mediated) transfer of a functional *UBE3A* transgene could potentially succeed in reinstating functional, long-lasting UBE3A expression in neurons with a single dose (21, 22). AAV-based gene transfer can also be relatively safe and effective, as evidenced by recent FDA approval for the treatment of CNS disorders including spinal muscular atrophy and inherited retinal disease (23, 24). Therefore, the paucity of efforts — clinical or preclinical — to leverage this technology toward the treatment of AS has been somewhat surprising. In fact, nearly a decade has passed since publication of the first and only study of AAV-mediated gene transfer in mice, which focused narrowly on targeted reinstatement of UBE3A in the adult hippocampus and realized commensurately limited phenotypic recovery (25). In the interim, studies modeling UBE3A reinstatement with the use of inducible genetic systems have clearly demonstrated the need for early intervention, as soon after birth as possible (14, 26). The benefits of brain-wide UBE3A reinstatement can be inferred from contemporaneous mouse studies probing the neural circuit basis of AS-relevant phenotypes (13, 27–29).

Here, we report on the toxicity and efficacy of a one-time intracerebroventricular (ICV) administration to neonatal AS model mice of a recombinant AAV9-derived PHP.B vector (30) encoding a codon-optimized human UBE3A (hUBE3Aopt) transgene. Through this approach, we model desirable features of AS gene therapy, achieving widespread hUBE3A reexpression in dorsal forebrain neurons, prior to the emergence of AS-like phenotypes and into adulthood. Furthermore, we establish the feasibility of reinstating dual-isoform hUBE3A expression, a benefit typically attributed only to paternal *UBE3A* unsilencing. Our work provides a rationale for future AS clinical trials based on hUBE3A gene transfer and offers a positive outlook for both symptom relief and the tolerance of hUBE3A overexpression.

## Results

### hUBE3Aopt yields traceable, physiological expression of short and long hUBE3A isoforms.

**Figure 1:**
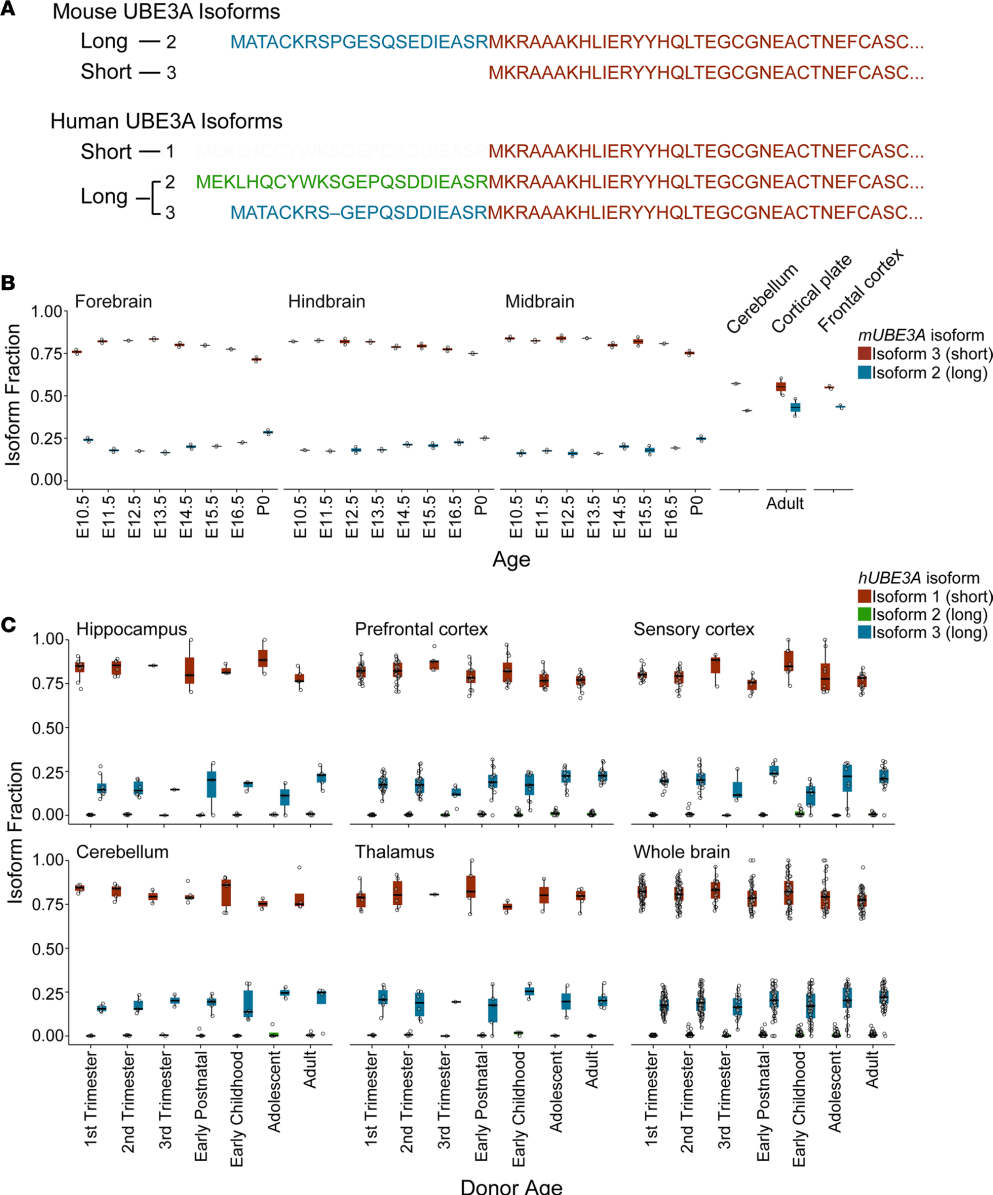
*UBE3A* isoform expression in mouse and human brain. (**A**) N-terminal amino acid sequences of human and mouse UBE3A isoforms. Highly conserved sequences beginning the short isoform are shown in burgundy; N-terminal amino acid extensions characteristic of long UBE3A isoforms are shown in blue or green. (**B**) Box plots of *mUbe3a* isoform expression in mouse brain regions across development. Whiskers represent 1.5 × interquartile range (IQR). Isoform fraction was calculated from RNA-seq coverage over exons common to all *mUbe3a* transcripts (exon 4) or specific to long *mUbe3a* (exon 3). The long *mUbe3a* (isoform 2) fraction was computed as Exon 3/Exon 4; the short *mUbe3a* (isoform 3) fraction was computed as (Exon 4 – Exon 3)/Exon 4. (**C**) Box plots of *hUBE3A* isoform expression in human brain regions across development and aging. Whiskers represent 1.5 × IQR. Isoform fractions were estimated from RNA-seq coverage over *hUBE3A* exons including exon 6, which is common to all *hUBE3A* transcripts, and exons 3 and 4, which encode long *hUBE3A* isoforms 3 and 2, respectively, when spliced to exon 6. Exon 3 and 4 reads were weighted according to exon 6 splicing frequencies previously published for human cortex (33). Long *hUBE3A* isoform 3 was computed as (0.322 × Exon 3)/Exon 6; long *hUBE3A* isoform 2 was computed as (0.071 × Exon 4)/Exon 6; short *hUBE3A* isoform 1 was computed as (Exon 6 – [(0.322 × Exon 3) + (0.071 × Exon 4)])/Exon 6.

Alternative splicing of mouse (*mUbe3a*) and human (*hUBE3A*) transcripts produces distinct UBE3A protein isoforms, which differ in their extreme amino N-termini (31). Short UBE3A isoforms, differentially numbered 3 in mouse and 1 in human, lack N-terminal extensions (of ~20 amino acids) common to their long isoform counterparts, of which there is a single representative in mice (isoform 2) and 2 in humans (isoforms 2 and 3) (Figure 1A). Noncoding mouse isoform 1 has no counterpart among human isoforms and was recently retracted from the NCBI database for insufficient evidence (32). Short UBE3A protein is reported to be expressed in excess of long UBE3A in both the mouse and human cortex at ratios of ~4:1 and ~3:1, respectively (32). To more thoroughly evaluate the proportionality of UBE3A isoform expression across space and time in the developing brain, we conducted a comprehensive survey using publicly available mouse and human RNA sequencing (RNA-seq) data. In mice, irrespective of brain region, we observed short/long *mUbe3a* expression ratios of nearly 4:1, consistent with previous findings for cortex during fetal development up until birth (32) (Figure 1B). By adulthood, *mUbe3a* short/long ratios declined to near 1:1, approaching a ~1:1.6 ratio recently reported for adult hippocampus (33) (Figure 1B). Human short/long *hUBE3A* ratios were also remarkably consistent (~4:1) across brain regions during development. However, unlike *mUbe3a* short/long ratios, they remained constant throughout aging, showing no decrease in the adult brain. Of the 2 long *hUBE3A* species, isoform 3 was far more abundant; isoform 2 expression fractions were near zero in many instances (Figure 1C and Supplemental Figure 1; supplemental material available online with this article; https://doi.org/10.1172/jci.insight.144712DS1). Collectively, these data support that short/long UBE3A isoform ratios are maintained with remarkable spatiotemporal consistency in the developing mouse and human brain.

**Figure 2:**
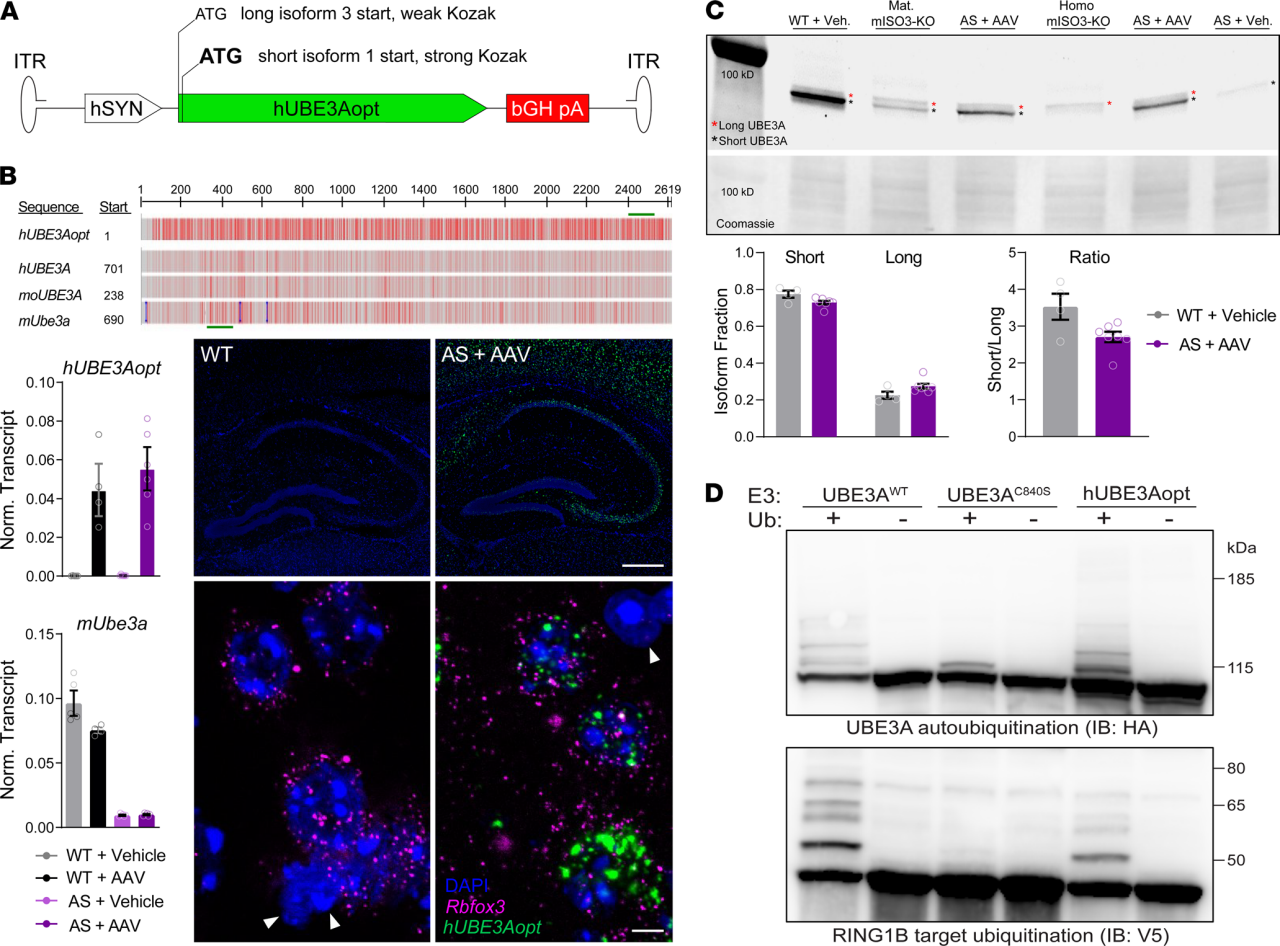
PHP. B/hUBE3Aopt yields traceable, dual-isoform hUBE3A expression. (**A**) PHP.B/hUBE3Aopt construct for expressing long (isoform 3) and short (isoform 1) isoforms of hUBE3A under control of a human synapsin promoter (hSYN). (**B**) Top: multiple sequence alignment of *hUBE3Aopt* with human (*hUBE3A*), NHP (*moUBE3A*), and mouse (*mUbe3a*) transcripts. Darker shades of red indicate greater sequence divergence. Green lines overlay *hUBE3Aopt* and *mUbe3a* amplicons in ddPCR assays. Bottom left: mean ± SEM transcript values (normalized to *Gapdh*) for *hUBE3Aopt* (top) and *mUbe3a* (bottom) in the brains of adult WT and AS mice following neonatal ICV administration of 1 μL of either vehicle or 1.6 × 10^14^ vg/mL PHP.B/hUBE3Aopt. Bottom right: representative HCR in situ hybridization labeling for *hUBE3Aopt* transcripts (green) in the neocortex and hippocampus of 1-month-old WT and AS + AAV mice (*n* = 3). Higher magnification depicts labeling in *Rbfox3*^+^ (magenta) neocortical neurons; arrowheads indicate nuclei of putative glia. Scale bars by level of magnification: 350 μm (low) and 5 μm (high). (**C**) Top: Western blotting of whole-brain lysates using a UBE3A antibody that binds mouse and human UBE3A equally, irrespective of isoform. Asterisks indicate the position of long (red) and short (black) UBE3A isoforms. Coomassie staining shows total protein loading. AS + AAV mice were administered 1 μL of 1.6 × 10^14^ vg/mL PHP.B/hUBE3Aopt as neonates. Homozygous mISO3-KO mice completely lack expression of short UBE3A, while mice with a maternally inherited deletion of isoform 3 (mat. ISO3-KO) lack neuronal expression of short UBE3A. Bottom: mean ± SEM fractional values of long and short UBE3A expression (left) and short/long isoform ratios (right). (**D**) Immunoblot demonstrating catalytic activity toward self (autoubiquitination) and RING1B (target ubiquitination) of different UBE3A constructs in an *E*. *coli*–based ubiquitination assay. Higher molecular weight ubiquitin-specific modifications were indicative of catalytic activity for WT UBE3A and hUBE3Aopt samples, but not samples expressing catalytically impaired UBE3A^C840S^. See supplemental material for unedited blots.

The evolutionary conservation of short/long UBE3A isoform ratios suggests that appropriate isoform representation will support the successful implementation of AAV-mediated *UBE3A* gene transfer. To devise an AAV vector capable of dual-isoform hUBE3A expression, we exploited the perfect alignment of short UBE3A amino acid sequences downstream of the long UBE3A N-terminal extensions (Figure 1A). This configuration enabled packaging of short *hUBE3A* isoform 1 and long *hUBE3A* isoform 3 (the most abundant long hUBE3A isoform) sequences contiguously within the same reading frame. Because each isoform is translated from an independent start codon, we differentially manipulated Kozak sequence strengths with the goal of favoring short hUBE3A (isoform 1) expression. The resulting expression cassette, termed hUBE3Aopt (Supplemental Table 1), further comprised regulatory sequences, including a human synapsin promoter for selective expression in neurons (Figure 2A). Codon optimization of hUBE3Aopt decreased base sequence identity with mice (74%), macaques (76%), and humans (76%) — a feature to be leveraged toward transgene tracing in recipient tissues (Figure 2B).

We packaged the hUBE3Aopt expression cassette into AAV9-derived PHP.B capsids to begin testing transgene expression properties in vivo, first pursuing ICV injections. Compared with intravascular administration, ICV injections, like other intracerebrospinal fluid routes of delivery, offer advantages in translational feasibility for CNS disorders: low doses that more readily scale from preclinical models to human, improved efficiency of CNS transduction, and the avoidance of circulating anti-AAV neutralizing antibodies (34–36). PHP.B yields robust CNS transduction via the ICV route, at least on par with the parent AAV9 capsid (37), but grants superior CNS access when delivered intravascularly in mice (30), offering greater flexibility for future preclinical studies. Following ICV delivery of 1 μL of 1.6 × 10^14^ vector genomes (vg)/mL of PHP.B/hUBE3Aopt vector to neonatal AS model mice, which come to lack neuronal expression of UBE3A (38), we performed quantitative PCR (qPCR) analysis of whole-brain RNAs. We successfully amplified *hUBE3Aopt* transcripts in PHP.B/ hUBE3Aopt-injected WT (WT + AAV) and AS (AS + AAV) mice but not vehicle-treated controls. In situ hybridization of *hUBE3Aopt* probes in AS + AAV brain tissues revealed a neuron-specific punctate signal that was absent from WT brains (Figure 2B), confirming that codon optimization enables selective detection of *hUBE3Aopt* among endogenous *mUbe3a* transcripts. In parallel, we conducted Western blotting analyses of whole-brain lysates to assess the capacity for *hUBE3Aopt* transcripts to be translated into both short and long hUBE3A isoforms. By comparison with samples from homozygous isoform 3 KO mice (mISO3-KO) that exclusively express long UBE3A, we were able to reliably identify bands belonging to both long and short hUBE3A in AS + AAV samples, thereby establishing that PHP.B/hUBE3Aopt is in fact a dual isoform-expressing vector. Quantification evinced a short/long expression ratio of 2.7:1, which was comparable with both the 3.5:1 ratio we obtained for WT + vehicle controls (Figure 2C) and a ~3:1 ratio previously determined for human cortical lysates (32). Importantly, when compared with hUBE3A encoded by WT expression constructs, hUBE3A proteins expressed from the *hUBE3Aopt* coding sequence proved similarly capable of autoubiquitination and substrate ubiquitination (Figure 2D). Thus, hUBE3Aopt yields traceable expression of both long and short hUBE3A in physiologically relevant proportions, and these proteins appear to be enzymatically competent.

### ICV injection of PHP.B/hUBE3Aopt results in widespread, developmentally dynamic hUBE3A expression in the brain.

**Figure 3:**
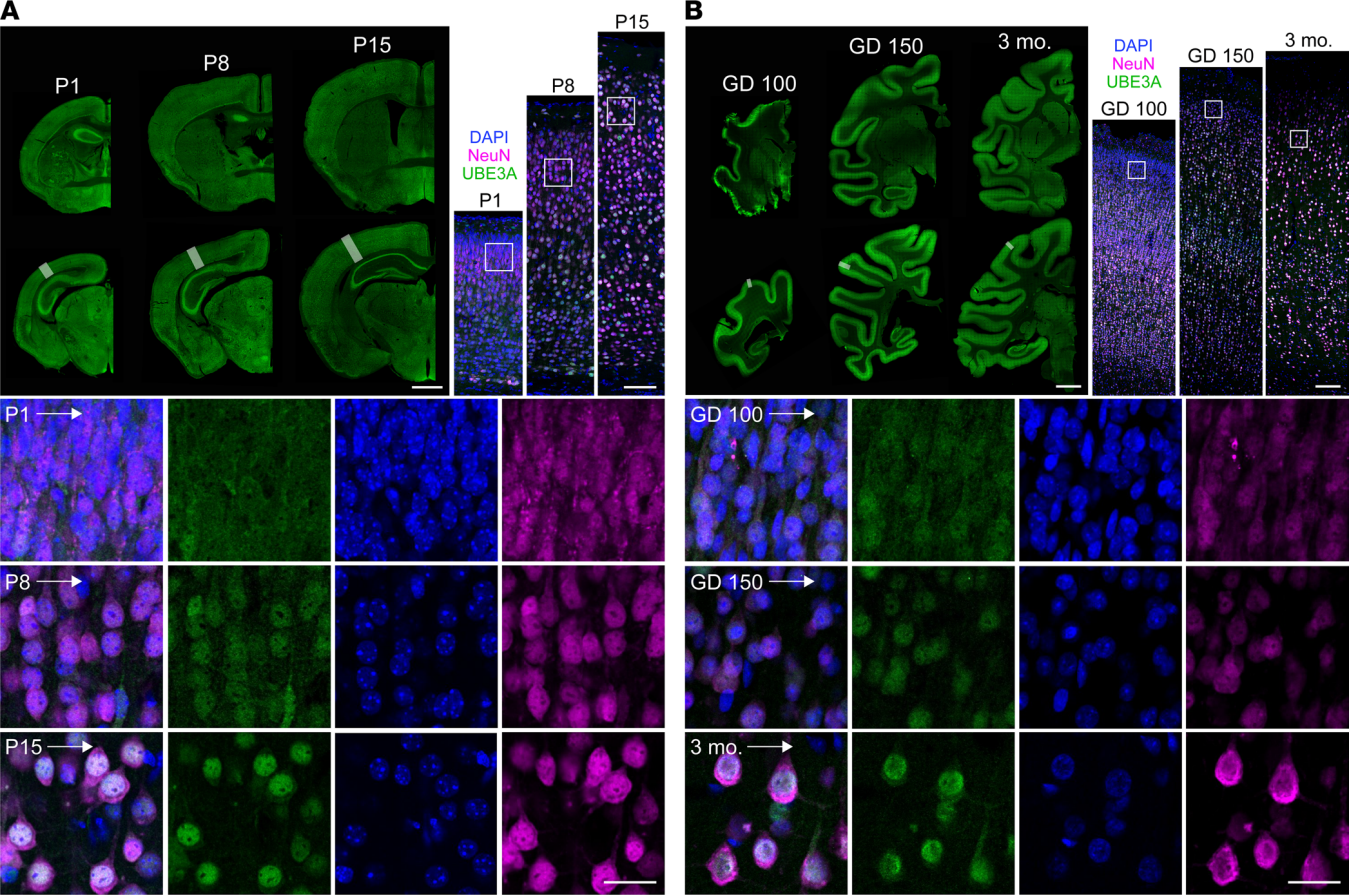
Conserved regional and subcellular expression of UBE3A in the developing mouse and NHP brain. (**A**) Representative images of UBE3A immunofluorescence staining in anterior (top row) and posterior (bottom row) coronal hemisections from the brains of P1, P8, and P15 mice (*n* = 2, each age). Higher-magnification images of boxed regions depict staining for UBE3A (green), DAPI (blue), and the neuronal marker NeuN (magenta) in strips of neocortex. Digital cropping of boxed regions in superficial cortex is displayed in rows below. (**B**) Representative images of UBE3A immunofluorescence staining in the brains of GD 100, GD 150, and 3-month-old rhesus macaque NHP (normal gestation is 165 days in rhesus monkeys), arrayed according to the layout in **A** (*n* = 2, each age). Scale bars by level of magnification: 1 mm (low), 85 μm (high), and 25 μm (cropped) (**A**); 5 mm (low), 150 μm (high), and 25 μm (cropped) (**B**).

Mouse modeling predicts that widespread neuronal UBE3A reinstatement, occurring before the closure of a critical early postnatal period (14, 26), will be maximally efficacious in the treatment of AS. Accordingly, UBE3A is expressed ubiquitously in the mouse nervous system from early stages of development onward (39, 40). To determine if UBE3A expression is similarly widespread in the nonhuman primate (NHP) nervous system, and to better define anatomical targets for hUBE3A gene transfer, we mapped UBE3A immunofluorescence in developmental coronal sections of the rhesus macaque brain. UBE3A staining was evident throughout the NHP brain at least as early as the end of the second trimester (gestational day 100; GD100), corresponding to the broad distribution observed in mouse at a similar developmental stage. Widespread UBE3A expression continued to be a shared feature of the developing mouse and NHP brain up until birth and through the early postnatal period (Figure 3, A and B). We also observed striking species similarities in staining at the subcellular level as development progressed — in particular, a cytoplasmic to nuclear shift in neuronal UBE3A labeling, which was previously documented in mice both in vitro and in vivo (32, 40, 41) (Figure 3, A and B).

**Figure 4:**
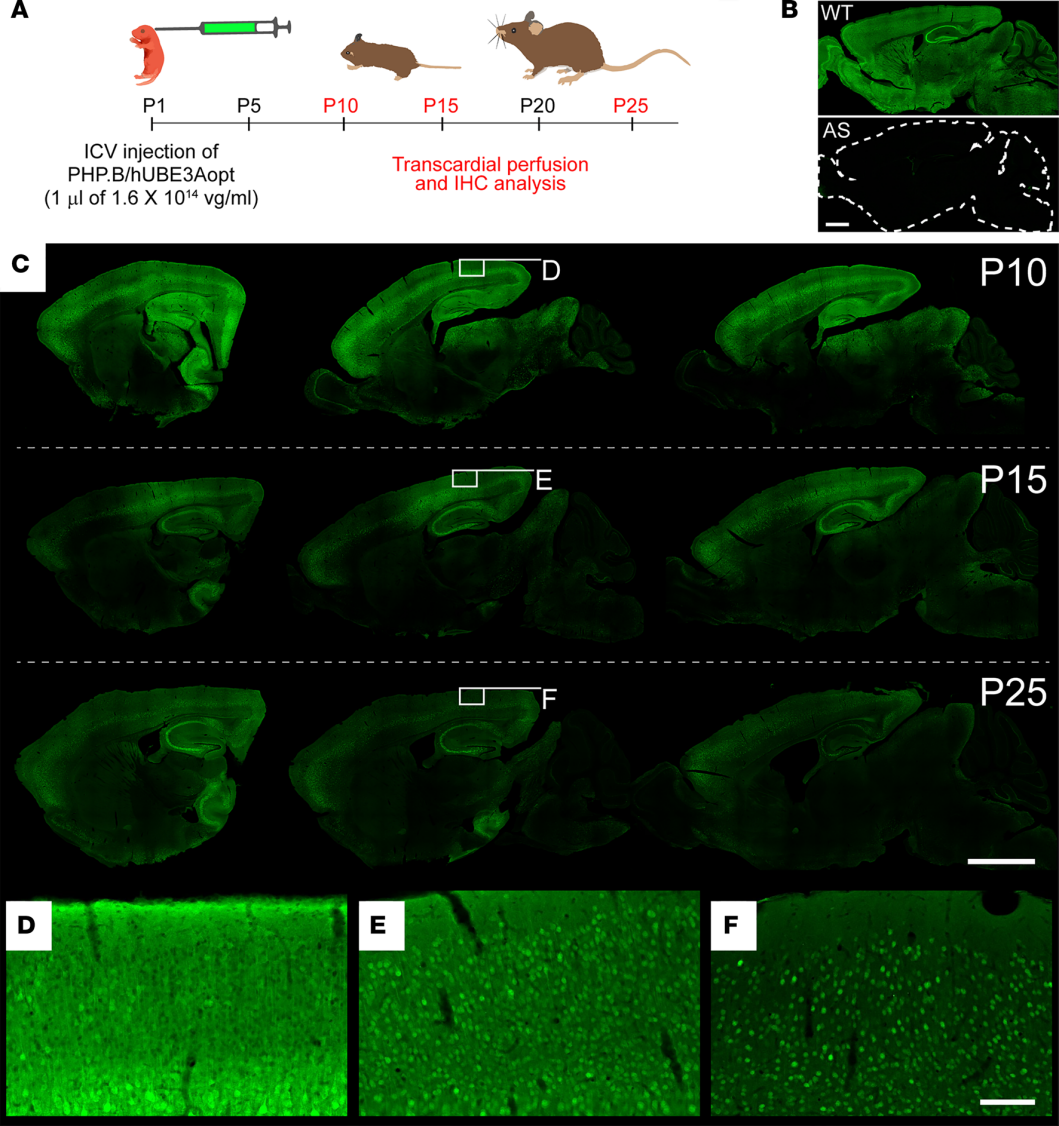
ICV injection of neonatal mice with PHP. B/hUBE3Aopt yields broad UBE3A reinstatement in dorsal forebrain neurons with fast onset and developmentally dynamic subcellular localization. (**A**) Schematic of experiment to evaluate hUBE3Aopt biodistribution in AS mice at various postnatal time points following neonatal ICV administration of 1 μL of 1.6 × 10^14^ vg/mL PHP.B/hUBE3Aopt. (**B**) Representative images of UBE3A immunofluorescence staining in adult WT and Angelman syndrome (AS) model mice. (**C**) Representative images of UBE3A expression in medial to lateral arrays of sagittal sections from P10 (top row), P15 (middle row), and P25 (bottom row) AS mice following neonatal ICV treatment as shown in **A** (*n* = 2, each age). (**D**–**F**) Higher-magnification images of boxed regions in **C**. Scale bars: 1.4 μm (**B**); 2 mm (**C**); 100 μm (**D**–**F**).

UBE3A subcellular localization in neurons is governed by interactions between *cis*-acting elements in specific isoforms and trans-acting protein partners like PSMD4, which — given the remarkable stability of UBE3A isoform expression ratios over development (Figure 1) — are themselves likely to be temporally dynamic in their expression (32). In mature mouse neurons, short UBE3A concentrates preferentially in the nucleus, whereas long UBE3A localizes nearly exclusively to the cytoplasm (Supplemental Figure 2) (32, 42). The scenario is similar for hUBE3A isoforms in mature human neurons, albeit with the least abundant long isoform (i.e., isoform 2) evolutionarily assuming the attribute of exclusive cytoplasmic localization and the most abundant long isoform (i.e., isoform 3) exhibiting a nuclear bias in its localization — akin to short hUBE3A (i.e., isoform 1) — despite its N-terminal sequence very closely matching that of the cytoplasmic long mouse isoform (Figure 1A) (33). Considering the hUBE3Aopt payload of isoforms 1 and 3, it followed that proteins expressed from this vector should faithfully organize in neuronal subcellular compartments according to endogenous patterns, coming to reflect a predominantly nuclear localization over time. We evaluated UBE3A immunofluorescence in AS mice at P10, P15, and P25 following neonatal ICV injection with PHP.B/hUBE3Aopt (Figure 4A). AS mice almost completely lack endogenous neuronal UBE3A (Figure 4B), enabling an uncompromised analysis of hUBE3A expression following gene transfer. Our efforts revealed hUBE3A labeling throughout the brain by P10, which was pronounced among neurons of the dorsal forebrain but relatively sparse in most midbrain and hindbrain structures. While this regional pattern persisted at P15 and P25 (Figure 4C), subcellular hUBE3A labeling changed dramatically, becoming less cytoplasmic and increasingly nuclear over time (Figure 4, D–F). Taken together, these findings establish that neonatal ICV injection of PHP.B/hUBE3Aopt reinstates UBE3A in the brains of AS mice with widespread distribution and rapid developmental onset, as well as according to native expression dynamics, which are all advantageous features of an AS gene therapy.

### ICV injection of PHP.B/hUBE3Aopt improves motor learning and the execution of innate behaviors in AS mice.

**Figure 5:**
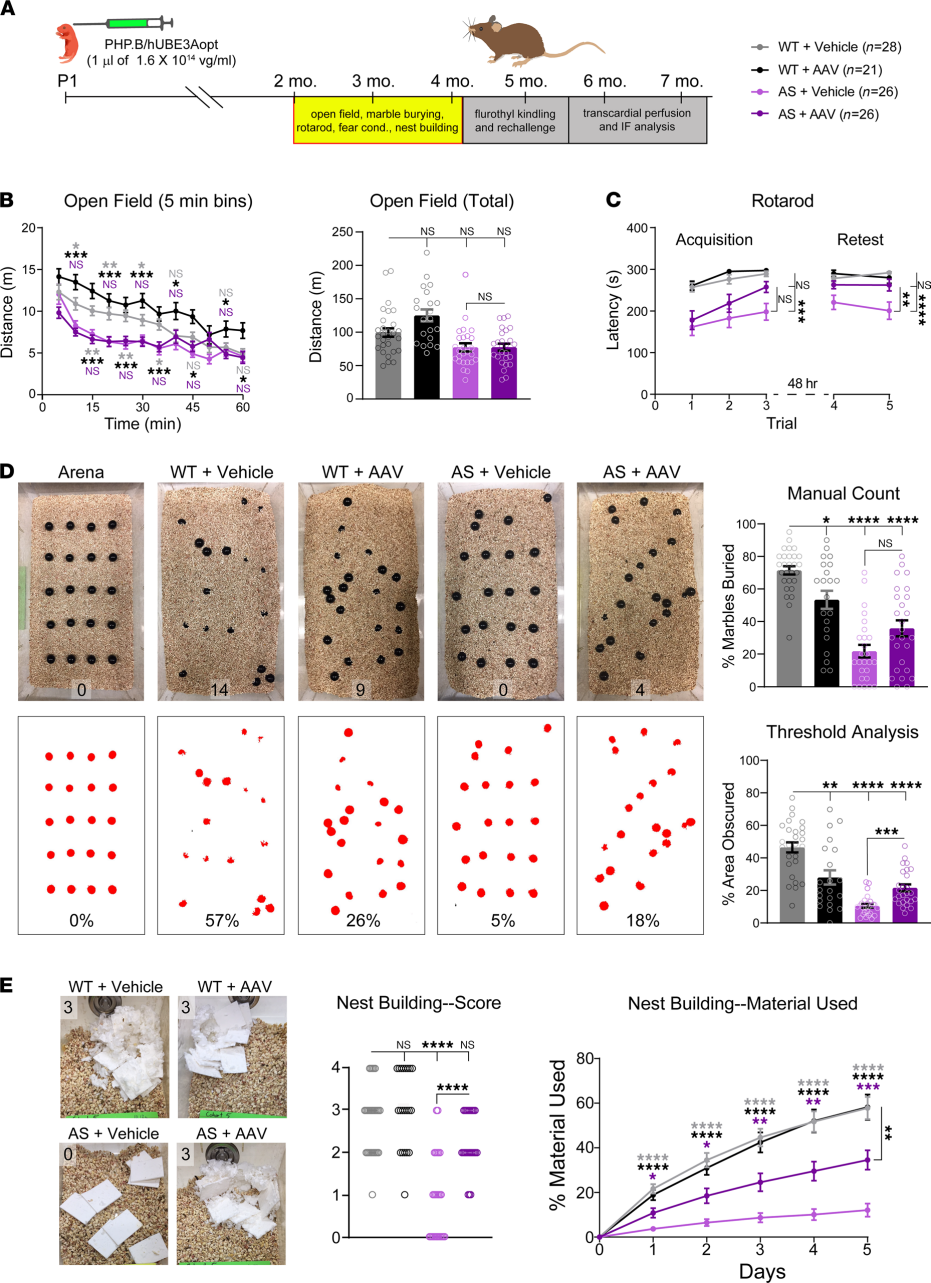
Neonatal ICV injection of PHP. B/hUBE3Aopt rescues motor learning and innate behaviors in AS mice. (**A**) Experimental timeline for evaluation of behavioral performance in adult AS mice following neonatal ICV administration of 1 μL of 1.6 × 10^14^ vg/mL PHP.B/hUBE3Aopt. Sample sizes for each experimental group are listed to the right. (**B**) Distance traveled in the open field, per 5 minutes (left panel) and total (right panel). Two-way repeated-measures ANOVA and 1-way ANOVA, Tukey’s post hoc. (**C**) Latency to fall off the accelerating rotarod during the acquisition and retest phases of the task. Two-way repeated-measures ANOVA, Tukey’s post hoc. (**D**) Top row: representative images of marble burying arenas before (far left) and following 30-minute test sessions. Percentage of marbles buried, as determined by manual counting, is plotted to the right. Bottom row: representative thresholded images of marble burying arenas before (far left) and following 30-minute test sessions. Percentage of marble area obscured by bedding is plotted to the right. Welch’s 1-way ANOVA, Dunnett’s post hoc. (**E**) Representative images of nests built by mice of each treatment group with inset scores for nest quality. Middle panel: graph of nest building scores. Kruskal-Wallis test followed by Mann-Whitney *U* post hoc with Bonferroni correction for multiple comparisons. Right panel: mean ± SEM nesting material used during the 5-day nest building assay. Two-way repeated-measures ANOVA, Tukey’s post hoc. Data are represented as means ± SEM except for nest building scores. *P < 0.05, **P < 0.01, ***P < 0.001, ****P < 0.0001.

To evaluate vector efficacy, we analyzed the behavioral performance of adult AS and WT littermate mice that were ICV-injected as neonates with 1 μL of either vehicle or 1.6 × 10^14^ vg/mL PHP.B/hUBE3Aopt. We administered a modified version of a behavioral test battery suitable for preclinical drug testing in AS model mice (43) (Figure 5A), replacing the forced swim test with flurothyl kindling to improve overall face validity for AS. Prior to beginning testing in mice 2.5–3.5 months of age, we commenced monthly body weight measurements. Body weight was equivalent between groups at 1 month, including in mice receiving PHP.B/hUBE3Aopt (WT + AAV and AS + AAV), thus indicating generally good tolerance of the treatment. Excessive adult weight gain has been well documented in the model (44, 45) and corresponds to observations of obesity in adult individuals with AS (46, 47). By 2 months, untreated AS mice (AS + vehicle) began to exhibit increased body weight relative to the WT groups. This effect was especially pronounced in the female AS + AAV group and strengthened statistically over the next 2 months of observation (Supplemental Figure 3), consistent with previous reports (19, 48). Although results were inconclusive for the male AS + AAV group, excessive adult weight gain was fully penetrant in female AS + AAV mice, indicating a lack of efficacy for PHP.B/hUBE3Aopt in normalizing this phenotype by early stages of adulthood (Supplemental Figure 3).

Motor dysfunction is a core feature of AS (1), which is reflected in the poor performance of AS mice on several behavioral tasks, including the open field and accelerating rotarod tests (43, 49). We lacked the statistical power to replicate an open field hypolocomotion phenotype in AS + vehicle mice when looking at aggregate performance (43), but we observed a clear deficit when analyzing the data by 5-minute bins. AS + vehicle and AS + AAV group performance was statistically indistinguishable, indicating that PHP.B/hUBE3Aopt failed to normalize open field activity in AS mice (Figure 5B). We next assessed rotarod performance, again finding evidence of baseline motor impairment in naive mice (trial 1) of both AS groups. However, whereas AS + vehicle performance remained relatively stable over successive trials, AS + AAV mice demonstrated increased learning during the acquisition phase, which they consolidated into significant motor improvements, enabling control-level performance during retest (Figure 5C).

AS mice have great difficulty executing species-typical behavioral routines such as digging and nest building (43). We used the marble burying task to evaluate the potential for PHP.B/hUBE3Aopt treatment to rescue deficits in digging behavior in AS mice. AS + AAV mice outperformed their AS + vehicle counterparts on marble burying, as was indicated by manual counts of buried marbles and statistically confirmed by unbiased image-based analysis. The group data comprised a broad range of individual responses: some AS + AAV mice attained WT + vehicle levels of performance, others failed to eclipse the spread of AS + vehicle values. Interestingly, we observed a very similar range of marble burying scores in the WT + AAV group (Figure 5D), raising the possibility that poor performance on this task resulted from hUBE3A overdosage. In contrast, group performances on the nest building task were more uniform. WT mice, regardless of treatment group, built robust nests incorporating most of the available nesting material over the 5-day testing period. AS + AAV mice fell short of matching WT levels of nest building, but they far surpassed the performance of AS + vehicle mice that consistently built nests of poor quality (Figure 5E). Overall, these findings support that neonatal gene transfer of hUBE3Aopt confers to AS mice a markedly increased capacity to learn motor tasks and express innate, species-typical behaviors.

### ICV injection of PHP.B/hUBE3Aopt mitigates exaggerated epileptogenesis and associated hippocampal pathology in AS mice.

Approximately 90% of individuals with AS also suffer from epilepsies that can be difficult to manage and that adversely affect quality of life (3). The spontaneous recurrent seizures that define epilepsy may exacerbate other phenotypes, including intellectual disability (50). Considering these clinical factors and the enrichment of hUBE3A expression in cortical and hippocampal circuits (Figure 4), we examined the potential for PHP.B/hUBE3Aopt treatment to rescue both cognitive deficits and seizure susceptibility in AS mice. Common learning paradigms in mice — such as Morris water maze and fear conditioning — expose rather mild and inconsistent deficits in the AS model that demand intensive statistical sampling (43). Accordingly, we obtained inconclusive results when fear conditioning adult WT and AS mice treated with PHP.B/hUBE3Aopt or vehicle as neonates (Supplemental Figure 4A). Although mice of each group clearly demonstrated associative learning of the unconditioned foot shock stimulus and conditioned auditory cue (Supplemental Figure 4B), we were underpowered to detect robust deficits in either contextual or cued fear memory in the AS + vehicle group — or rescue thereof (Supplemental Figure 4, C and D).

**Figure 6:**
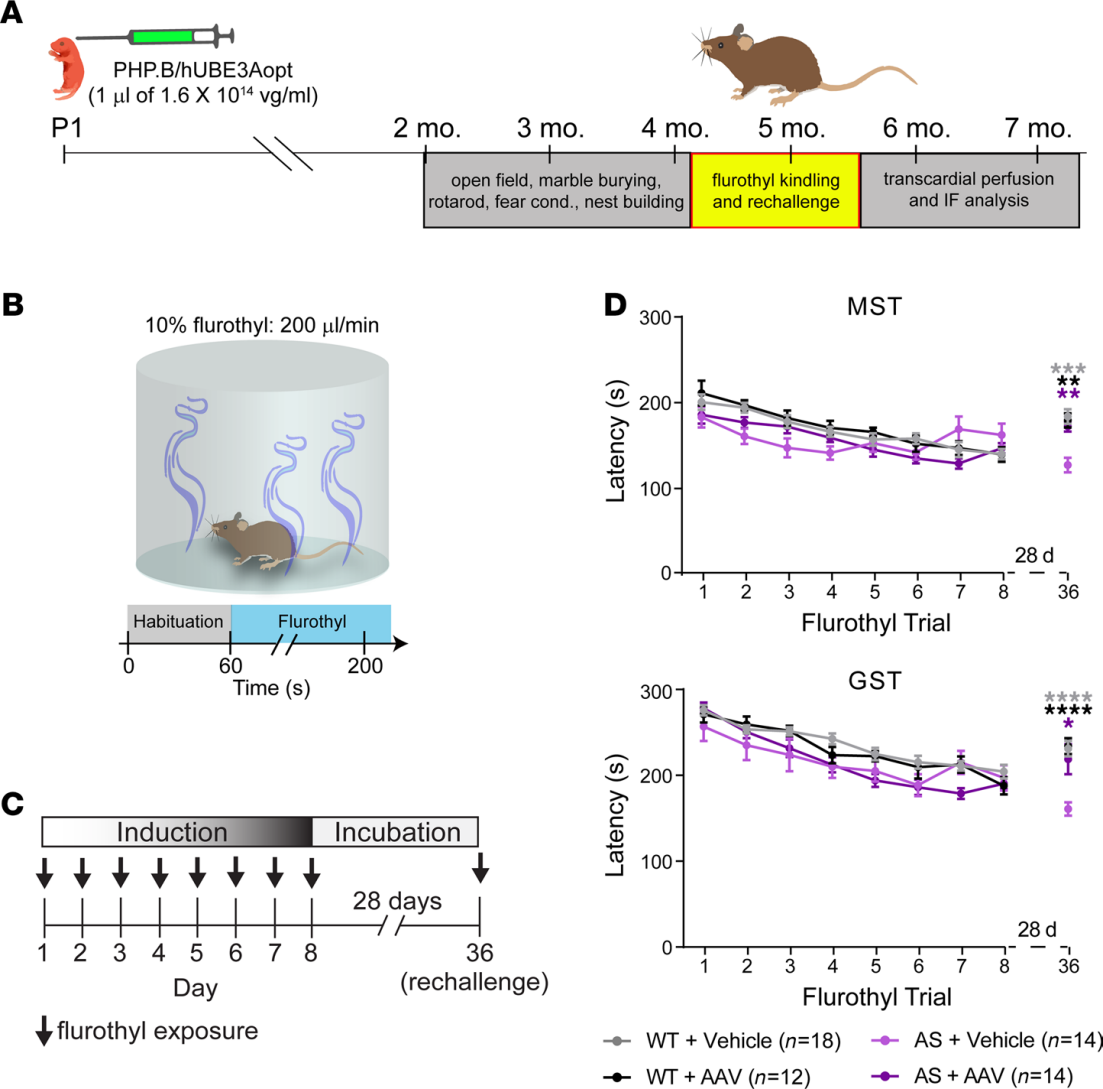
Neonatal ICV injection of PHP. B/hUBE3Aopt mitigates seizure susceptibility following flurothyl kindling in AS mice. (**A**) Experimental timeline for evaluation of behavioral seizure responses to flurothyl kindling and rechallenge. (**B**) Schematic of flurothyl-induced seizure protocol. (**C**) Schematic of experimental paradigm for 8-day flurothyl seizure kindling and rechallenge. (**D**) Graphs of mean ± SEM latencies to myoclonic (top panel) and generalized seizure (bottom panel) depicting changes in seizure threshold (MST, myoclonic seizure threshold; GST, generalized seizure threshold) in response to flurothyl kindling and rechallenge. Two-way repeated-measures ANOVA, Tukey’s post hoc. Data are shown as mean ± SEM. *P < 0.05, **P < 0.01, ***P < 0.001, ****P < 0.0001.

Increased susceptibility to acute audiogenic seizure induction is a long-established, highly reproducible phenotype of AS mice (43). We more recently demonstrated enhanced epileptogenic potential in the model using the flurothyl kindling and rechallenge paradigm (29). One week following completion of the nest building assay (Figure 6A), we subjected PHP.B/hUBE3Aopt- and vehicle-treated mice to the first of 8 once-daily seizure induction trials with flurothyl (Figure 6B), a volatile GABA_A_ receptor antagonist (51). Seizure latencies decreased over the kindling phase of the protocol in all groups, resulting in convergent myoclonic seizure thresholds (MST) and generalized seizure thresholds (GST) by day 8 (Figure 6, C and D). However, following a prolonged 28-day period without flurothyl, rechallenge with a single exposure showed WT and AS + AAV group thresholds that had rebounded toward day 1 group levels, while AS + vehicle thresholds were even lower than their day 8 kindled values (Figure 6, C and D). These results generally confirm our previous finding that AS mice exhibit long-term, heightened sensitivity to seizures following flurothyl kindling (29). Rescue of enhanced rechallenge sensitivity in AS + AAV mice suggests that PHP.B/hUBE3Aopt treatment may have the capacity to mitigate related proepileptogenic phenotypes in AS mice.

**Figure 7:**
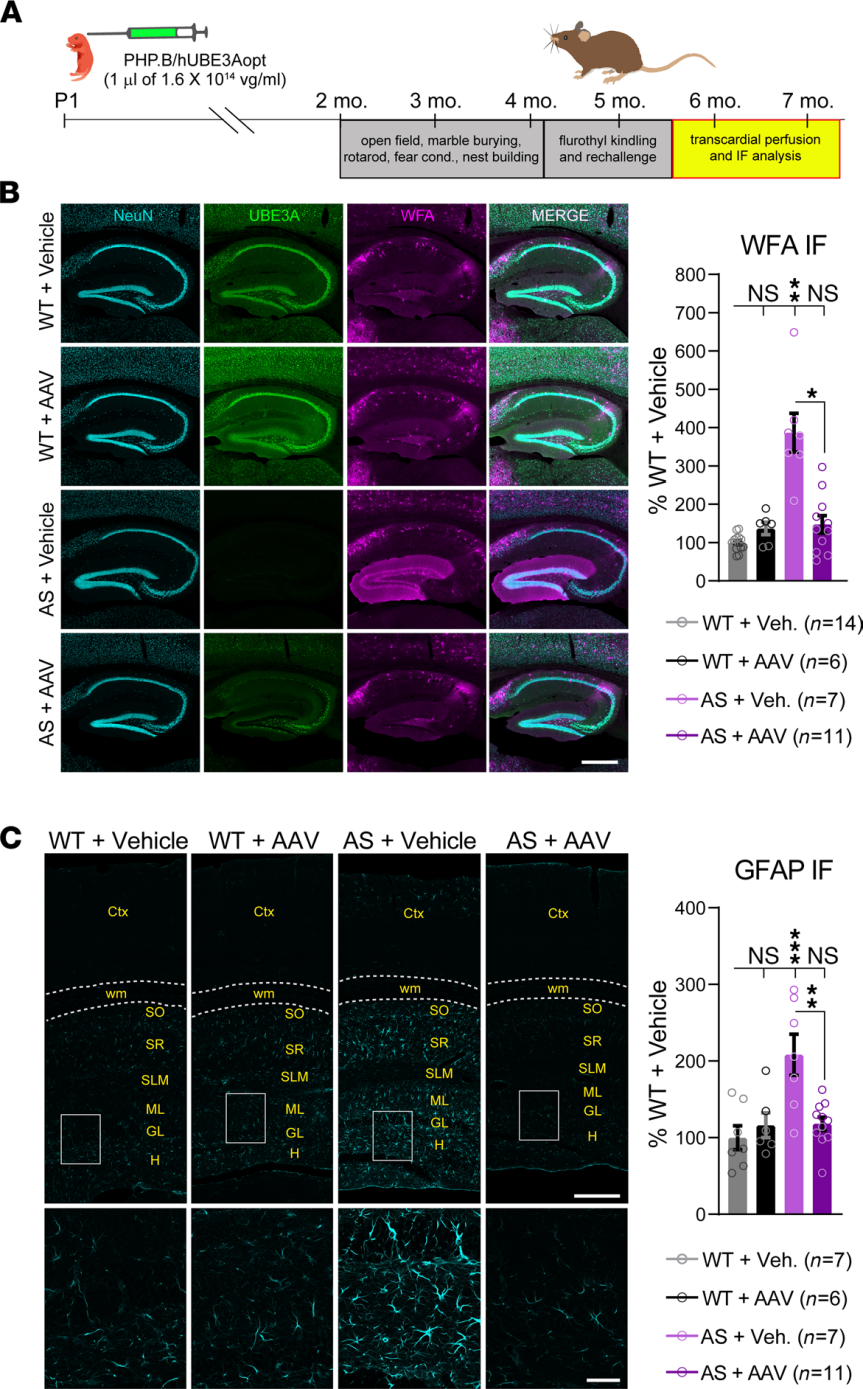
Neonatal ICV injection of PHP. B/hUBE3Aopt protects against flurothyl kindling–induced hippocampal pathology in AS mice. (**A**) Experimental timeline for immunofluorescence analysis following flurothyl seizure kindling. (**B**) Representative images of *Wisteria floribunda* agglutinin (WFA) staining of perineuronal nets (magenta) in sagittal sections of the dorsal hippocampus. Costaining for NeuN (cyan) and UBE3A (green) is also displayed. Scale bar: 500 μm. Right panel: quantification of normalized mean WFA fluorescence in the dentate gyrus. Welch’s 1-way ANOVA, Dunnett’s post hoc. (**C**) Representative images of glial fibrillary acidic protein (GFAP) staining in sagittal sections of the dorsal hippocampus and cortex. Ctx, cortex; WM, white matter; SO, stratum oriens; SR, stratum radiatum; SLM, stratum lacunosum moleculare; ML, molecular layer; GL, granule cell layer; H, hilus. Scale bars by level of magnification: 250 μm (low) and 50 μm (high). Right panel: quantification of normalized mean GFAP fluorescence in the hippocampus. One-way ANOVA, Tukey’s post hoc. Data are shown as mean ± SEM. *P < 0.05, **P < 0.01, ***P < 0.001.

Hippocampal pathology is a hallmark of temporal lobe epilepsy (52–54) and often occurs in mice following the induction of seizures (55, 56). We previously identified a striking, albeit atypical, histopathological correlate of enhanced epileptogenic potential in AS mice revealed by flurothyl kindling: aberrant deposition of perineuronal nets (PNNs) in the dentate gyrus, evidenced by dramatically increased staining for *Wisteria floribunda* agglutinin (WFA) (29). We therefore transcardially perfused flurothyl-kindled mice with 4% paraformaldehyde within an hour of flurothyl rechallenge to facilitate a series of post hoc histological analyses, including WFA staining (Figure 7A). Compared with WT mice from either treatment group, brain sections from AS + vehicle mice displayed intense WFA staining that concentrated in the molecular layer of the dentate gyrus. Despite modestly elevated levels at baseline (Supplemental Figure 5), WFA staining in the dentate gyrus of AS + AAV mice was statistically similar to WT following kindling and rechallenge, in keeping with their normal behavioral seizure responses (Figure 6D and Figure 7B). The PNN phenotype in flurothyl-kindled AS + vehicle mice was suggestive of broader hippocampal pathology, so we also investigated the potential for reactive astrogliosis via analysis of GFAP immunofluorescence. Typically, flurothyl-kindled mice on the C57BL/6J background are impervious to this insult (57); that we observed robust GFAP immunofluorescence throughout the hippocampus of flurothyl-kindled AS + vehicle mice, in the absence of any elevation in GFAP levels prior to flurothyl exposure (Supplemental Figure 5B), is reflective of their exaggerated epileptogenesis. In contrast, AS + AAV mice exhibited WT levels of hippocampal GFAP immunofluorescence in response to flurothyl kindling, indicating rescue of this phenotype, as well (Figure 7C). Collectively, these data demonstrate the potential for neonatal gene transfer of hUBE3Aopt to improve seizure outcomes associated with AS.

### Postmortem analysis of hUBE3A expression following ICV injection of PHP.B/hUBE3Aopt.

**Figure 8:**
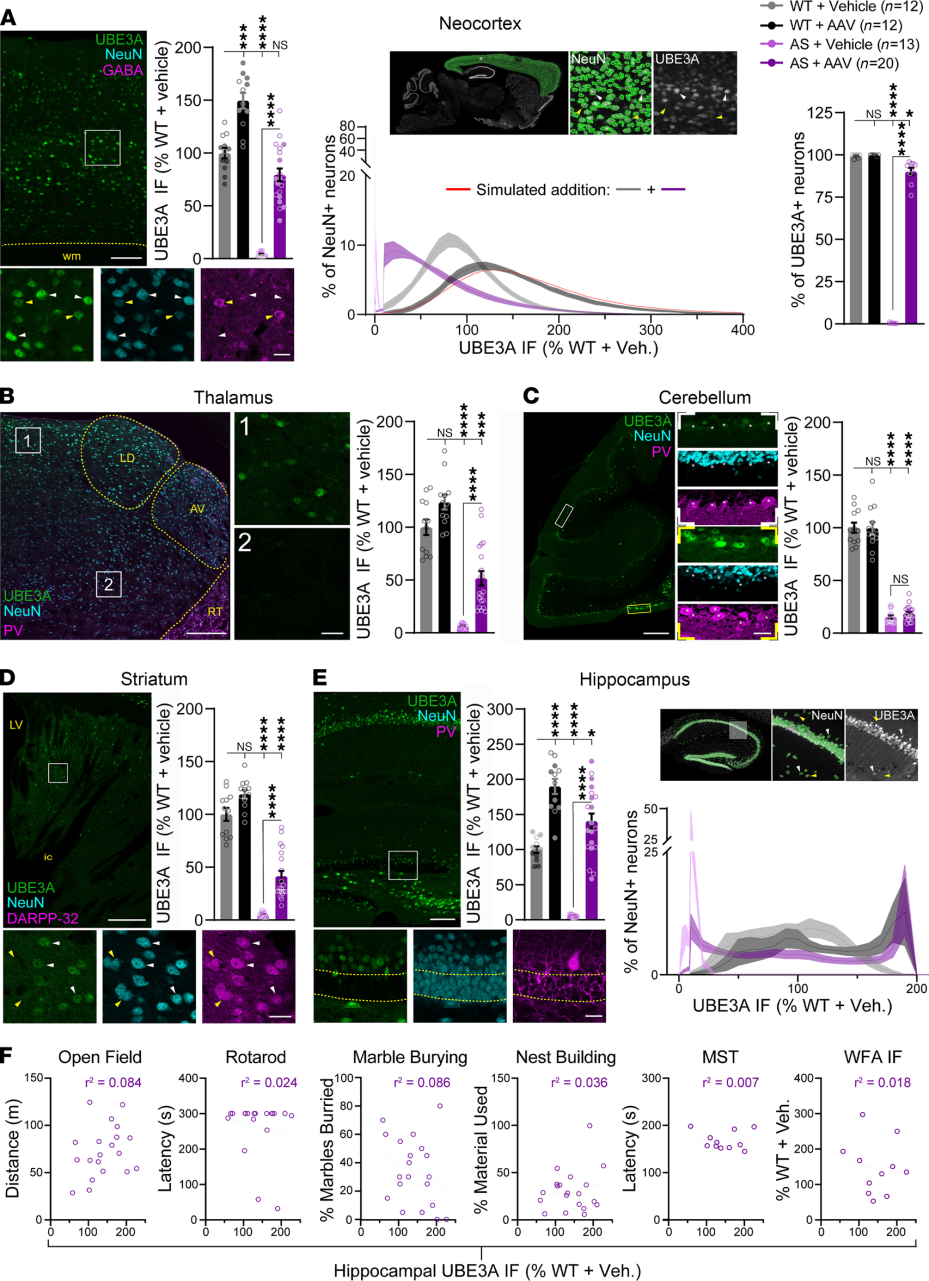
PHP. B/hUBE3Aopt-mediated UBE3A overexpression is largely restricted to the hippocampus and does not correlate with phenotypic outcome. (**A–E**) Mean ± SEM UBE3A immunofluorescence (IF) by brain region. *P < 0.05, ***P < 0.001, ****P < 0.0001. Welch’s ANOVA, Dunnett’s *post hoc*. (**A**) Left: neocortical UBE3A IF, counterstaining for NeuN and GABA. Arrowheads indicate putative excitatory neurons (white) and GABAergic interneurons (yellow). Middle: representative NeuN-based segmentation of neurons with (white arrowheads) or without (yellow arrowheads) UBE3A IF. Graphed distribution of UBE3A IF levels within individual neurons for each group (>44,000 neurons analyzed per animal, from a subset of animals corresponding to filled circles in mean IF graph). Red line depicts the distribution resulting from the simulated addition (*n* = 50,000 per simulation; 10 simulations) of randomly selected WT + vehicle and AS + AAV values. Right: quantification of the percentage of NeuN^+^ neurons costained for UBE3A. (**B**) Thalamic UBE3A IF, counterstaining for NeuN and parvalbumin (PV). (**C**) Cerebellar UBE3A IF, counterstaining for NeuN and PV. (**D**) Striatal UBE3A IF, counterstaining for NeuN and DARPP-32. Arrowheads indicate medium spiny neurons with (white) or without (yellow) UBE3A labeling. (**E**) Left: hippocampal UBE3A IF, counterstaining for NeuN and PV. Right: representative NeuN-based segmentation of neurons with (white arrowheads) or without (yellow arrowheads) UBE3A IF. Graphed distribution of UBE3A IF levels within individual neurons for each group (>2300 neurons analyzed per animal, from a subset of animals corresponding to filled circles in mean IF graph). (**F**) Pearson’s *r* correlation of normalized mean hippocampal UBE3A IF and phenotypic outcome in AS + AAV mice. LV, lateral ventricle; LD, lateral dorsal nucleus; AV, anteroventral nucleus; RT, reticular nucleus; wm, white matter; ic, internal capsule. Scale bars by level of magnification: 100 μm (low), 20 μm (high) (**A** and **E**); 250 μm (low), 30 μm (high) (**B**); 200 μm (low), 30 μm (high) (**C**); 200 μm (low), 20 μm (high) (**D**).

Dup15q syndrome is an autism spectrum disorder (ASD) caused by duplications and triplications of the 15q11.2–q13.1 chromosomal region that encompasses *UBE3A* and other genes (20). hUBE3A overexpression is therefore of particular concern for gene transfer approaches to treating AS. To investigate the levels and biodistribution of hUBE3A expression in our study, we surveyed UBE3A immunofluorescence across major brain structures in AS + AAV mice (Figure 8, A–E, and Supplemental Figure 6). We found UBE3A immunofluorescence to be widespread in the neocortex, generally inclusive of both excitatory and inhibitory neurons in AS + AAV subjects. AS + AAV mean neocortical UBE3A levels were statistically indistinguishable from WT, indicating infrequent hUBE3A overexpression; just 2 of 20 of the evaluated AS + AAV neocortices exceeded 116% of the WT + vehicle mean. hUBE3A underexpression was more common in this group, as demonstrated by a strongly left-skewed distribution of UBE3A immunofluorescence measured from individual neocortical neurons. In fact, 9.1% ± 2.26 % of AS + AAV neurons showed a lack of detectable UBE3A immunofluorescence, compared with just 0.73% ± 0.37 % of WT + vehicle neurons — evidence that hUBE3A may have failed to express at all in a modest, but significant, subset of these targeted neocortical cells. In WT + AAV mice, neocortical UBE3A overexpression was prevalent, with mean immunofluorescence ~150% of WT + vehicle levels. Further analysis revealed this overexpression to be the simple summation of hUBE3A and endogenous WT UBE3A expression, as the distribution of neuronal UBE3A immunofluorescence in WT + AAV neocortex nearly matched the simulated distribution resulting from summation of UBE3A levels from pairs of neocortical neurons randomly chosen from the AS +AAV and WT + vehicle groups (Figure 8A). This disputes the notion that autoubiquitination prevents UBE3A overexpression risk in gene transfer applications (25).

Evidence of hUBE3A overexpression was scarce in the thalamus (Figure 8B), cerebellum (Figure 8C), and striatum (Figure 8D) of AS + AAV mice. On average, mean UBE3A immunofluorescence in AS +AAV thalamus and striatum was 50% of WT + vehicle levels, characterized by distinct gradients of expression, with neurons nearest to the ventricles most likely to stain for UBE3A (Figure 8, B and D). Mean UBE3A immunofluorescence in AS + AAV cerebellums was very low — statistically indistinguishable from average WT + vehicle values — due to sparse and patchy hUBE3A expression (Figure 8C).

hUBE3A expression was evenly distributed across most subregions of AS + AAV hippocampi, with the exception of the inner half of the dentate gyrus granule cell layer, in which it was notably absent (Figure 8E). The neurons populating this subregion were likely not yet born at the time of PHP.B/hUBE3Aopt injection (58); their neural progenitors may have been poorly transduced or failed to survive following transduction. The hippocampus was the only brain structure in which we consistently observed hUBE3A overexpression in AS + AAV mice. Nearly all subjects in the group exhibited hippocampal UBE3A immunofluorescence in excess of WT + vehicle values. hUBE3A overexpression ranged from 160% to 225% in the most extreme half of cases, but this was likely an underestimate: AS + AAV and WT + AAV distributions of neuronal UBE3A immunofluorescence were cut off at apparent maxima of ~200% of WT + vehicle levels, a consequence of saturated signal in a significant proportion of cells (Figure 8E).

To begin to explore the potential functional impact of hippocampal hUBE3A overexpression, we performed correlation analyses of hippocampal UBE3A immunofluorescence and phenotypic outcomes in AS + AAV mice. Hippocampal hUBE3A levels were not predictive of performance on any behavioral task or histopathological measure of a priori interest (Figure 8F and Supplemental Figure 4, C and D). However, further histological analysis of AS +AAV mice revealed dentate gyrus atrophy in hippocampi with the greatest degree of hUBE3A overexpression (Supplemental Figure 7A). The dentate gyrus lesion was usually hemispherically asymmetrical (Supplemental Figure 6 and Supplemental Figure 7B), signaling that it resulted from parenchymal mistargeting of ICV injections. We observed a strikingly similar linear relationship in WT + AAV mice (versus AS + AAV slope: *P* = 0.64), albeit significantly rightward-shifted (versus AS + AAV elevation: *P* < 0.0001). Succinctly, equivalent UBE3A immunofluorescence values associated with a significantly lesser degree of dentate gyrus atrophy in WT + AAV versus AS + AAV mice (Supplemental Figure 7A), suggesting that this lesion was not a consequence of hUBE3A overexpression per se, but rather a result of general toxicity owed to the vector. This was confirmed through control PHP.B/hSYN-EGFP injections in which hippocampal EGFP overexpression proved to be similarly predictive of dentate gyrus atrophy (Supplemental Figure 7, D and E). Asymmetrical dentate gyrus volume loss possibly hampered our ability to evince rescue of reduced brain weight in AS + AAV mice (Supplemental Figure 8) — a phenotype with high face validity for microcephaly in AS (2, 44, 59, 60) — since the normalization of AS + AAV brain weight showed a significant negative correlation with the degree of dentate gyrus atrophy. (Supplemental Figure 7C).

## Discussion

This proof-of-principle study establishes neonatal gene transfer of hUBE3A as a potentially viable approach to treating AS. The prelude to this preclinical endeavor was characterization of the isoform specificity and developmental biodistribution of UBE3A in the mouse and primate (nonhuman and human) brain. In so doing, we revealed 3 remarkably well-conserved UBE3A expression features across species: (a) highly consistent ratios of short to long isoform expression during brain development; (b) dynamic subcellular localization in neurons, whereby UBE3A becomes increasingly nuclear as maturation progresses; and (c) ubiquitous neuronal expression with early developmental onset. These parameters guided our design of a potentially novel dual-isoform hUBE3Aopt vector, in which we manipulated Kozak sequence strengths to bias expression in favor of short UBE3A, successfully recapitulating ~3:1 short/long protein isoform representation. hUBE3A gene transfer now shares with paternal UBE3A unsilencing approaches (15–17) the capacity to match nuanced aspects of endogenous isoform expression, which may be necessary to reinstate the full repertoire of neuronal UBE3A functions resident to specific subcellular compartments.

A growing literature supports that hUBE3A reinstatement should ideally be administered during the early postnatal period for maximal therapeutic effect in AS (61). Indeed, neonatal ICV injection of PHP.B/hUBE3Aopt aptly rescued innate behaviors (e.g., nest building) whose windows for treatment by UBE3A reinstatement close extremely early in AS mice, within 2–3 weeks of birth (14). This treatment resulted in hUBE3A expression with sufficiently early onset to improve innate behavioral responses in AS mice, so it was not surprising that we also evinced efficacy in the treatment of both rotarod deficits and exaggerated epileptogenesis, which remain amenable to genetic UBE3A reinstatement up to 4–6 weeks after birth (14). In contrast, the open field phenotype in AS mice was refractory to PHP.B/hUBE3Aopt, despite having a similar, possibly even broader, treatment window (14). PHP.B/hUBE3Aopt treatment also failed to improve baseline performance on the rotarod in AS mice. We surmise that a factor besides the timing of treatment was at play in these instances — likely poor hUBE3A expression in subcortical motor areas. If so, this portends limited efficacy for hUBE3Aopt in treating other behavioral deficits rooted in subcortical circuitry unless vector biodistribution is optimized. Intravascular delivery of PHP.B can be harnessed to model improved subcortical transduction in AS mice (30), but evidence of much poorer biodistribution in NHP brains indicates that this capsid has limited translatability to the treatment of AS individuals (62–64). Combinatorial testing for capsids and delivery routes that maximize widespread targeting of the NHP brain is essential and will be facilitated by the eminent traceability of the codon-optimized hUBE3Aopt vector in recipient tissues, which we have demonstrated here.

Ideally, technical advances that improve the biodistribution of hUBE3Aopt in the brain will not come at the price of hUBE3A overexpression, which is almost certainly a pathogenic contributor to Dup15q syndrome. *UBE3A* is the only 15q11.2–q13.1 gene with exclusively maternal monoallelic expression in neurons, and Dup15q syndrome is most commonly associated with maternally inherited 15q11.2–q13.1 duplications and triplications (20). The neurodevelopmental impact of *UBE3A* overexpression apart from that of the other ~20 overexpressed genes in this chromosomal region is unknown, but familial cases of circumscribed maternal *UBE3A* duplication have shed some light on the matter. Compared with individuals with Dup15q syndrome, individuals with selective *UBE3A* duplication had favorable neurodevelopmental outcomes characterized by a spectrum of variably diagnosed, relatively mild neuropsychiatric phenotypes, including developmental delay, autistic features, depression, and anxiety (65).

In our study, we seldom observed hUBE3A overexpression outside of the hippocampus. The hippocampus protrudes into the ventricular space and so would seem particularly vulnerable to PHP.B/hUBE3Aopt overdosage via ICV injection. However, hippocampal hUBE3A overexpression was typically asymmetrical, leading us to conclude that inadvertent parenchymal targeting was the cause of this undesirable outcome and could be avoided with improved technique — in the laboratory and certainly in the clinic (66). Importantly, this expression abnormality did not correlate with behavioral performance and so presumably did not bear on our capacity to mark improvements in motor learning, nest building, and seizure outcome in AS mice. hUBE3A levels did strongly correlate with hippocampal atrophy that was disproportionately localized to the dentate gyrus in treated AS mice. This suggested that dentate granule cells or their progenitors might be particularly sensitive to toxic effects of hUBE3A overexpression, especially as it occurs with rapid onset following gene transfer. However, neither *Ube3a*-overexpressing mice nor individuals with Dup15q syndrome exhibit such severe lesions of the dentate gyrus (9, 67–69). More importantly, the relationship between hUBE3A overexpression and dentate gyrus lesions proved not to be causal. We confirmed this through control injections of PHP.B/hSYN-EGFP, whereby hippocampal EGFP overexpression resulted in a similar lesion. As was recently shown, AAV internal terminal repeat sequences are themselves sufficient to mediate dose-dependent toxicity and cell death in the hippocampus, preferentially in dentate gyrus neural progenitor cells (70). This is a plausible mechanism to explain the dentate gyrus lesions we observed in our study and should serve as a cautionary tale for gene therapy approaches that expose the hippocampal neurogenic niche to high doses of AAV.

Our study has certain limitations. First, the timing of treatment in our study is difficult to translate clinically. The neonatal period in mice corresponds to late second trimester development in humans. After factoring in a lag from AAV transduction to vector expression, we had likely reintroduced hUBE3A protein to the brain by P7, a time point equivalent to full-term human gestation (71). Typically, AS diagnoses are predicated on the presentation of salient clinical features beginning at 6 months of age (1), so based on current diagnostic practices, our study is idealistic with respect to modeling early intervention.

Second, we have not yet determined whether reintroducing both short and long hUBE3A to the developing nervous system is advantageous to reintroducing short hUBE3A alone. AS mouse phenotypes tested to this point have proven selectively sensitive to short UBE3A deletion (32) and are therefore ill suited to gauge the added therapeutic benefit that reexpression of long UBE3A may provide. Experiments pitting dual-isoform vectors against vectors expressing short hUBE3A alone are warranted once long UBE3A-sensitive phenotypes have been confirmed. Recent work defining the electrophysiological maturation of human embryonic stem cell–derived neurons in the wake of selective long hUBE3A isoform deletion is encouraging in this regard (72). Despite the current lack of empirical evidence, the circumstantial case for pursuing dual-isoform hUBE3A vectors for AS gene therapy is compelling. Short and long UBE3A expression has been conserved over the course of placental mammalian evolution (64) — a span of greater than 100 million years — implying important, distinctive developmental roles. These roles may depend on isoform-specific access to different subcellular compartments — the mechanisms of which continue to be elucidated (33, 72) — and unique protein complexes. Clinically, start codon mutations that selectively abrogate expression of short hUBE3A manifest in a less severe form of AS (73). These rare cases strongly suggest that, while short hUBE3A is critical for supporting neurotypical development, coexpression of long hUBE3A isoforms contributes to an optimal developmental outcome.

Third, our vector design currently provides for expression of only 1 of 2 possible long hUBE3A isoforms. We chose to include isoform 3 because it is far more abundant than isoform 2, but isoform 2 may play a key developmental role despite its low expression levels. Isoform 2 arose in NHP species prior to the deletion of an isoform 3 N-terminal proline residue that dramatically shifted its localization to the nucleus. This sequence of evolutionary events prompted the hypothesis that the proline deletion was tolerated only because isoform 2, which is exclusively cytoplasmic in cultured neurons, was able to assume cytoplasmic functions vacated by isoform 3 (33). Based on proteomic analyses, isoform 2 likely participates in some unique protein-protein interactions within the cytoplasm, but overall, its protein partners are highly redundant with those of isoform 3 (74). Moreover, although isoform 2 is itself strictly localized to the cytoplasm, it does not have exclusive access to this compartment among hUBE3A isoforms; we clearly observed hUBE3Aopt to mediate developmentally appropriate patterns of cytoplasmic hUBE3A localization with expression of isoforms 1 and 3 alone.

Fourth, we administered treatment to mice in the absence of any overt behavioral training or environmental stimulation — 2 factors sure to be experienced by recipients of gene transfer therapy in the course of a clinical trial and that could synergize with the synaptic plasticity unlocked by hUBE3A reinstatement to increase learning capacity (75). Thus, our studies might be underestimating the potential for behavioral recovery in AS model mice, and future experiments should incorporate environmental enrichment and learning-based tasks to better capture the full therapeutic potential of hUBE3A gene transfer. But just as mouse models may underestimate the capacity for hUBE3Aopt to prevent or reverse AS phenotypes, they may similarly fail to fully expose the potential for hUBE3A overexpression to disrupt neurological function in patients. This could be especially true for higher-order cognitive abilities and social behaviors that are not well represented in mice in the first place. Nevertheless, given the link between UBE3A overexpression and ASD (76), and our observation that hUBE3Aopt was linked to impaired marble burying in WT mice, rigorous tests of cognitive performance and sociability in mice following AAV-mediated hUBE3Aopt transduction should be pursued in future studies.

Long-term CNS impacts of hUBE3Aopt treatment must be thoroughly considered to establish its safety profile and, by extension, its translational viability. As noted above, UBE3A-linked ASDs indicate at least some measure of neurodevelopmental risk associated with UBE3A overexpression. However, these disorders are unlikely to lend insight into late-onset neurotoxicities that could result from extreme instances of UBE3A overexpression. These may be rooted in biased AAV-mediated delivery among neuronal subpopulations, which may vary as a function of capsid type, administration route, and developmental age (77–81). Emphasizing this point is the recent discovery of toxic gain of function resulting from prolonged overexpression of survival motor neuron protein (SMN) in the course of gene therapy for spinal muscular atrophy (78). Mechanistically, as an E3 ubiquitin ligase with transcriptional coactivating functionality (7), UBE3A might be expected to significantly disrupt protein homeostasis and gene transcription if chronically overexpressed. Accordingly, it will be prudent to pursue proteomic and transcriptomic profiling of nervous system tissues in follow-up safety studies — preferably with single-cell resolution (82, 83) — even in the absence of neurotoxic effects leading to overt synaptic loss and neurodegeneration in the aftermath of hUBE3Aopt treatment. Beyond mouse studies, long-term safety must be further demonstrated in NHPs, especially as novel capsids and alternative routes of delivery are pursued to improve biodistribution. NHP safety studies will provide an especially stringent evaluation of potential adverse events due to UBE3A overexpression, with transgenic UBE3A expressed in addition to endogenous protein upon hUBE3Aopt treatment in neurotypical monkeys.

Finally, clinical biomarkers for AS must be further developed. Enhanced delta rhythmicity has emerged as a promising, noninvasive electroencephalographic biomarker of forebrain circuit disruption in both AS mice and individuals with AS (84). It will be critical to determine if delta rhythmicity is sensitive to UBE3A reinstatement — hUBE3Aopt-mediated or otherwise — since this physiological readout would greatly facilitate monitoring of treatment responses in future clinical trials.

## Methods

Supplemental methods are available online with this article. All chemicals used in this study were manufactured by Sigma-Aldrich or Thermo Fisher Scientific.

### Study design.

The goal of this study was to assess in AS model mice the toxicity and efficacy of a recombinant AAV9-PHP.B vector encoding long and short protein isoforms of hUBE3A (PHP.B/hUBE3Aopt). Neonatal (P0.5–P2) AS mice and WT littermate controls were randomly assigned to treatment with vehicle or PHP.B/hUBE3Aopt at a dose of 1.6 × 10^11^ vg delivered bilaterally to the cerebral ventricles. A subset of treated mice was set aside for experiments to validate expression of hUBE3A transcript and protein. All other treated mice (*n* = 101) were subjected to a battery of behavioral tests beginning at 10–14 weeks of age, spanning a period of 4 months, administered in the following order: open field, marble burying, rotarod, fear conditioning, and nest building. Most treated mice (*n* = 69) also underwent an additional flurothyl kindling and rechallenge analysis. Upon the completion of behavioral experiments, precisely 1 hour following flurothyl rechallenge when applicable, mice were sacrificed for histological analyses of protein expression. Numbers of experimental mice were based on previous behavioral studies of the same AS mouse model in which rigorous statistical power analyses were performed (43). No animals were excluded from study, and no data were excluded from analysis. All investigators who conducted experiments or collected and analyzed data were blinded to genotype and treatment until completion of the study.

### Mice.

Mice carrying a *Ube3a*-KO allele were originally generated in the laboratory of A. Beaudet (38) and back-crossed to a congenic C57BL/6J background (RRID:IMSR_JAX:016590). We generated maternal *Ube3a*-deficient mice (*Ube3a*^m−/p+^, also referred to as AS model mice) by crossing congenic C57BL/6J WT males to paternal *Ube3a*-deficient females (*Ube3a*^m+/p−^), which themselves are phenotypically normal (38, 85). Mice carrying an allele incapable of *Ube3a* isoform 3 expression (ISO3-KO mice; ref. 32) were maintained on a congenic C57BL/6J background. All mice in this study were raised on a 12:12 light-dark cycle with ad libitum access to food and water. Male and female littermates were included at equivalent genotypic ratios.

### AAV vector construction and production.

A codon-optimized human UBE3A cDNA, hUBE3Aopt, was designed using a commercially available algorithm tool (Atum). hUBE3Aopt encodes both hUBE3A isoform 1 and hUBE3A isoform 3, which are translated from independent start codons within a single reading frame. Kozak sequences were modified to enhance production of short hUBE3A isoform 1 (CAGGATGA) relative to long hUBE3A isoform 3 (TTTTATGG). The hUBE3Aopt cDNA was subcloned into plasmid pTRs-KS-hSYN-GFP-BGHpA to generate pTRs-KS-hSYN-hUBE3Aopt-BGHpA. pTRs-KS-hSYN-GFP-BGHpA was, itself, the product of subcloning a human synapsin promter (hSYN) into plasmid pTRs-KS-CBh-GFP-BGHpA (National Gene Vector Biorepository).

hUBE3Aopt was packaged into AAV9-PHP.B capsids (30), using methods developed by the UNC Gene Therapy Center Vector Core facility (86). Purified PHP.B was dialyzed in PBS supplemented with 5% D-sorbitol and an additional 212 mM NaCl (350 mM NaCl total). Vector titers were determined by qPCR (87) and confirmed by polyacrylamide gel electrophoresis (PAGE) and silver staining. PHP.B/hUBE3Aopt vg were single stranded.

PHP.B/hSYN-EGFP vectors were produced by HHMI-Janelia Viral Tools using a polyethyleneimine triple-transfection protocol in 293 cells grown under serum-free conditions. Vectors were purified through 3 rounds of CsCl density gradient centrifugation, each carried out for 16 hours at 8°C. The initial round was performed at 65,000 rpm (*g* = 341,650 average; 401,747 max), and the subsequent 2 rounds were done at 90,000 rpm (*g* = 560,196 average; 645,019 max). Purified vectors were exchanged into storage buffer containing 1× PBS, 5% sorbitol, and 350 mM NaCl.

### Vector delivery.

P0.5–P2 mouse pups were cryoanesthetized on wet ice for 3 minutes; they were then transferred to a chilled stage equipped with a fiber optic light source for transillumination of the lateral ventricles. A 10 μL syringe fitted with a 32-gauge, 0.4-in-long sterile syringe needle (7803-04, Hamilton) was used to bilaterally deliver 0.5 μL of purified PHP.B/hUBE3Aopt vector (1.6 × 10^14^ vg/mL) to the ventricles, resulting in a total dose of 1.6 × 10^11^ vg injected per pup. The addition of Fast Green dye (1 mg/mL; Sigma-Aldrich) to the virus solution facilitated the visualization of successful injections. Following injection, pups were warmed on an isothermal heating pad with home-cage nesting material before being returned en masse to their nursing dam.

### Data and materials availability.

All data associated with this study are available in the main text or the supplemental materials.

### Statistics.

One-way ANOVA followed by Tukey’s post hoc test was performed for open-field, WFA immunofluorescence analysis in naive mice, and GFAP immunofluorescence analysis was performed in kindled mice. Welch’s 1-way ANOVA followed by Dunnett’s post hoc test was carried out for marble burying, WFA immunofluorescence analysis was performed in kindled mice, GFAP immunofluorescence analysis was performed in naive mice, and quantification of UBE3A immunofluorescence was performed by brain region, as the Brown-Forsythe test indicated unequal variances in each case. To analyze ordinal nest building scores, the Kruskal-Wallis test was used, followed by Mann-Whitney *U* post hoc testing with Bonferroni correction for multiple comparisons. Two-way repeated-measures ANOVA, followed by Tukey’s post hoc test was performed for rotarod testing, nest building (material used), fear conditioning, and flurothyl kindling assays. In the figures, all values are expressed as means ± SEM unless otherwise specified. A *P* value less than 0.05 was considered significant. GraphPad Prism 8.3 software (GraphPad Software, RRID:SCR_002798) was used for statistical analyses.

### Study approval.

Animal studies followed NIH guidelines and were performed in strict compliance with animal protocols approved by the IACUC of the UNC at Chapel Hill.

## Author contributions

MCJ designed and performed experiments, analyzed data, and wrote the manuscript. CS designed and validated AAV vectors. JMS designed and performed bioinformatic analyses. CRD performed and analyzed behavioral studies. AMP designed and performed *E*. *coli*–based ubiquitination assays. MTS performed and analyzed immunofluorescence staining experiments and analyses. NWM performed in situ hybridization experiments. KDR designed and generated PHP.B/hSYN-EGFP vectors. YE contributed to experimental design and advised on bioinformatic analyses. DGA contributed to experimental design. SJG oversaw AAV vector production and designed experiments. BDP helped design experiments and contributed to data analysis. All authors edited the manuscript.

## Supplementary Material

Supplemental data

Supplemental table 1

## Version 1 10/22/2021

Electronic publication
