# Supplementary material for: Dual-isoform hUBE3A gene transfer improves behavioral and seizure outcomes in Angelman syndrome model mice
Source: JCI Insight. 2021 Oct 22;6(20):e144712. doi: 10.1172/jci.insight.144712 (PMC8564914; doi:10.1172/jci.insight.144712)
Supplement: Supplemental data [file jciinsight-6-144712-s170.pdf]

Supplemental Methods

Calculation of Ube3a/UBE3A isoform fractions from public RNA-seq data

Mouse ENCODE RNA-seq alignment bigWig files for developmental timepoints (E10.5 - P0) were downloaded from encodeproject.org under project series ENCSR505AHT, ENCSR328UYN, and ENCSR443OEA for midbrain, hindbrain, and forebrain, respectively. Alignment files corresponding to adult tissues were similarly downloaded for experiments ENCSR000BZM, ENCSR000BZR, and ENCSR000BZS for cerebellum, cortical plate, and frontal cortex, respectively. Signal covering the common exon 4 (chr7:59247173-59247217) and the isoform 2-specific exon 3 (chr7:59243350-59243469) (mm10 reference) was tabulated using deeptools multiBigwigSummary. Isoform fractions were computed in R as follows:

Isoform 2 (long) fraction = Exon 3/Exon 4

Isoform 3 (short) fraction = (Exon 4 – Exon 3)/Exon 4

Human RNA-seq data summarized to exons were obtained from BrainSpan (http://www.brainspan.org/static/download.html), and filtered for *UBE3A* exons as annotated by Gencode v10. Expression values were averaged over broader brain areas and developmental epochs. Brain areas included hippocampus (HIP), prefrontal cortex (DFC, VFC, MFC, OFC), sensory cortex (S1C, A1C, M1C, M1C-S1C), cerebellum (CB, CBC), and thalamus (DTH, MD). Developmental epochs were defined as follows: 1st trimester (8–13 postconceptional weeks (pcw)), 2nd trimester (16–25 pcw), 3rd trimester (26–37 pcw), early postnatal (4 months–2 years), early childhood (3–11 years), adolescent (13–19 years), and adult (19–40 years). Isoform fractions were estimated from RNA-seq coverage over exon 6 (chr15:25650607-25650653), which is common to all *hUBE3A* transcripts, and exons 3 (chr15:25654234-25654354) and 4

(chr15:25653766-25653795), which encode long *hUBE3A* isoforms 3 and 2, respectively, when spliced directly to exon 6. Exon 3 and 4 reads were weighted according to exon 6 splicing frequencies previously published for human cortex (1), and incorporated into the following equations:

Isoform 3 (long) fraction = (0.322*Exon 3)/Exon 6

Isoform 2 (long) fraction = (0.071*Exon 4)/Exon 6

Isoform 1 (short) fraction = (Exon 6 – ((0.322*Exon 3) + (0.071*Exon 4)))/Exon 6

Exon numbering was according to mouse and human isoform 2 as previously reported (1). Error bars for mouse RNA-seq data represent the standard deviation in isoform fraction. Error bars for human RNA-seq data represent 95% confidence intervals from 10,000 bootstrap samples of the data with replacement, and were computed in R using the rsample package. Bar plots and boxplots were generated in R version 3.6 and plotted with ggplot2. All code to reproduce these figures is available at https://github.com/jeremymsimon/Judson_UBE3A.

ddPCR detection of codon-optimized hUBE3A transcripts

Droplet digital polymerase chain reaction (ddPCR, QX200 platform, Bio-Rad, Hercules, CA) was used for qPCR analysis of mRNA transcript levels. Total neocortical RNAs were extracted using the RNeasy Mini Kit (Qiagen, Germantown, MD), and reverse transcribed via iScript random priming (Bio-Rad). 2 ng of the resulting cDNAs constituted the input for ddPCR assays designed to specifically assess levels of endogenous *Ube3a* and vector-derived *hUBE3Aopt* mRNAs. *Ube3a*-specific (Forward: tgaacaagaaggaaggaaaaga; Reverse: caattcctcctttgtgtgctg; Probe: FAM-ccgaaagctcagaaccagtgcctcagca-IowaBlack) and *hUBE3Aopt*-specific (Forward:

tgttcctccaattcaccacc; Reverse: cacgttgaaacaagtgtggg; Probe: FAM-ccccagtcctccgactggcgct-IowaBlack) primer/probe sets were each multiplexed with a primer/probe set for the *Gapdh* reference transcript (Forward: attgtggaagggctcatgac; Reverse: atgcagggatgatgttctgg; Probe: HEX-cgccacagctttccagaggggcca-IowaBlack) to facilitate the relative quantification of transcript levels. Primers were prepared at a final concentration of 900 nM, probes at 250 nM, and reactions were run using ddPCR Supermix for probes (Bio-Rad, 1863026).

In situ hybridization

Hybridization Chain Reaction (HCR, Molecular Instruments, Los Angeles, CA) was used to visualize multiple cellular RNA targets in fresh frozen tissues. The *Rbfox3* probe set was designed against transcript variant 1 (NM_001039167.1). A custom *hUBE3Aopt* probe set was designed against a target region spanning 223-2434 bp of the *hUBE3Aopt* vector coding sequence (**Table S1**). Briefly, brains were rapidly harvested via dissection and snap-frozen in dry ice-cooled isopentane prior to sectioning on a cryostat to a thickness of 20 µm. HCR was performed according to manufacturer specifications.

Western blotting analysis

Approximately 15 µg of total protein lysate, extracted from one whole cerebral hemisphere per sample, was loaded onto a 4-15% polyacrylamide gel and separated by SDS-PAGE. Electrophoresed proteins were transferred onto nitrocellulose membranes, which were subsequently blocked with Odyssey blocking buffer (LI-COR, Lincoln, NE, 927-50000) for 1 h at room temperature. Blocked membranes were incubated at 4 °C overnight with primary

antibodies diluted in a 1:1 solution of blocking buffer and Tris-buffered saline containing 0.1% Tween-20 (TBST). Primary antibodies included mouse anti-UBE3A (Sigma, 1404508, RRID:AB_10740376), diluted 1:1,000. Membranes were then washed repeatedly with TBST prior to incubation for 1 h at room temperature with secondary antibodies prepared in the same diluent as the primary antibodies. Secondary antibodies included 800cw donkey anti-mouse IgG (LI-COR, 926-32212), diluted 1:7,500. At the end of the incubation, membranes were washed repeatedly with TBST, followed by 3 final washes with TBS, immediately preceding analysis of infrared fluorescence using an Odyssey CLx imager (LI-COR).

E. coli in vivo ubiquitination assay

The intrinsic catalytic activity of hUBE3Aopt was assessed with a previously established *E. coli*-based ubiquitination assay (2, 3). BL21-GOLD (DE3) [*B* F^{-} *ompT hsdS*(r_b^{-} m_b^{-}) *dcm*$^{+}$ *Tet*^{r} *gal λ(DE3) endA Hte*] cells were co-transformed with three separate constructs encoding E1-E2-Ub, HA-tagged UBE3A variants—wild-type, C840S, and hUBE3Aopt—and V5-RING1B. Single transformants were grown at 37°C overnight in LB medium supplemented with 2% glucose, 50 mM Tris pH 8 and antibiotics as needed. On the next day, the cultures were diluted to an OD600 of 0.2, and grown at room temperature until an OD600 of 0.7 was reached. At this point, overnight protein synthesis was induced with 0.5 mM Isopropyl β-D-1-thiogalactopyranoside (IPTG, Sigma-Aldrich I6758) at a temperature of 16 °C. The cells were ultimately collected in 20 OD600-units, which were sonicated for 6 x 20 s at 5 mAmp in lysis buffer (50 mM Na-Pi buffer (pH 8.0), 300 mM NaCl, 5% glycerol, 100 µl PMSF (17.4 mg/ml), 100 µl Protease Inhibitor Cocktail (4.28 mg), 10 µl DNase (10 mg/ml), 10 µl RNase (10 mg/ml), 3.5 µl 2-Mercaptoethanol. Finally, the protein equivalent of 0.3 OD units per sample was loaded onto

SDS-PAGE gels and analyzed by immunoblotting. Horseradish peroxidase (HRP)-conjugated antibodies against HA (Roche, Basel, Switzerland, 12013819001, RRID:AB_390917) and V5 (Thermo Fisher Scientific, R961-25, RRID:AB_2556565) were used for probing HA-UBE3A and V5-RING1B, respectively.

Behavioral testing and analysis

Open field: Locomotor activity in a novel environment was assessed in a photocell-equipped automated chamber (41 cm × 41 cm × 30 cm; Versamax system, Accuscan Instruments). Activity chambers were contained inside sound-attenuating boxes equipped with ceiling-mounted lights and fans. Total distance traveled and rearing movements were recorded over the course of a 1 h trial.

Marble burying: Mice were tested in a clean cage located in a sound-attenuating chamber with ceiling light and fan. The cage contained 3 L of corncob bedding (Andersons Lab, 1/8 in diameter, irradiated), approximately 5 cm deep. 20 black glass marbles (14 mm diameter) were arranged on top of the bedding in an equidistant 5 × 4 grid. Animals were given access to the marbles for 30 min. At the end of the trial, mice were carefully removed from the cage, and the number of buried marbles (i.e., at least 50% covered by bedding) was recorded. In addition, overhead photographs of the testing arena were taken at the start and end of each trial, from which the percentage of marble area obscured by bedding was digitally computed using ImageJ software (RRID:SCR_003070) (4).

Rotarod: Mice were tested for balance and motor coordination on an accelerating rotarod (UgoBasile, Stoelting Co., Wood Dale, Il). Revolutions per minute (rpm) were set at an initial

value of 3, with a progressive increase to a maximum of 30 rpm across 5 min, the maximum trial length. 3 consecutive trials were administered to each mouse during the acquisition session. 48 hr later, two additional trials were conducted during the retest session. Intertrial intervals were 3-5 min. Latency to fall off the rotarod, or until the occurrence three consecutive wrapping/passive rotations, was recorded.

Fear conditioning: The Near-Infrared image tracking system (MED Associates, Burlington, VT) was used to conduct the fear-conditioning procedure across 3 days. On the first day, the training session, mice were placed in the test chamber, contained in a sound-attenuating box, and allowed to explore for 2 min to establish baseline levels of mobility and immobility. Mice were then exposed to a 30-s tone (80 dB), followed by a 2-s foot shock (0.4 mA). Mice received 2 additional shock-tone pairings, with 80 s intertrial intervals. Increased freezing during successive 30-s tone presentations was interpreted as learning of the shock-tone association. Context-dependent learning was evaluated on the second day of testing. Mice were placed back into the original test chamber, and levels of freezing (immobility) were determined across a 5-min session. On the third day of testing, mice were evaluated for associative learning of the auditory cue in a final 5-min session, which took place in chambers modified to obscure the original testing context: plexiglass inserts were added to change the wall and floor surface, and a novel odor (vanilla flavoring) was added to the sound-attenuating box. After 2 min of habituation to the novel context, the acoustic stimulus was presented for a 3-min period, wherein levels of freezing were analyzed.

Nest building: Mice were single-housed for a period of 3 to 5 days before the start of the experiment. On day 1, used nesting material provided by the animal care facility was replaced with 11 ± 1 g of compressed extra-thick blot filter paper (Bio-Rad, 1703966), cut into 8 evenly

sized rectangles. The amount of paper not incorporated into a nest was weighed and noted each day, for 5 consecutive days. On the final day of the assay, mice were carefully removed from their cages and overhead photographs of their nests were taken for *post hoc* analysis of nest quality, assessed according to a 5-point ordinal scale adapted from a previous study (5): 0=no nest, obvious neglect of nesting material; 1=rudimentary nest, minimal incorporation of nesting material; 2=disorganized nest, partial incorporation of nesting material; 3=organized nest, defined borders, but somewhat flat with incomplete incorporation of nesting material; 4= "crater-like" nest, well-defined vertical structure, near complete incorporation of nesting material.

Flurothyl kindling: Mice were habituated for 1 min in a lidded 2-L glass chamber prior to infusion of 10% flurothyl (bis-2,2,2-trifluoroethyl ether; Sigma-Aldrich) in 95% ethanol at a rate of 200 μL/min onto a disk of filter paper (Whatman, Grade 1) suspended at the top of the chamber. Myoclonic (sudden involuntary jerk/shock-like movements involving the face, trunk, and/or limbs) and generalized seizures (clonic-forebrain seizures characterized by clonus of the face and limbs, loss of postural control, rearing, and falling) were carefully monitored. Upon the emergence of a generalized seizure, the lid of the chamber was immediately removed, allowing for rapid dissipation of the flurothyl vapors and exposure of the mouse to fresh air. Mice were then returned to their home-cage following recovery from behavioral seizures. Between trials, the flurothyl chamber was recharged with fresh filter paper, cleaned using 70% ethanol, and thoroughly dried. For kindling, flurothyl exposures were repeated once daily over eight consecutive days (i.e., the induction phase). Mice were then given a 28-day rest period in their home-cages (i.e., the incubation phase), prior to receiving a final flurothyl re-exposure (i.e., rechallenge). Each flurothyl trial was video-recorded and reviewed by investigators who determined latency to the onset of myoclonic and generalized seizures.

Tissue collection and processing

Nonhuman primates: Pre- and postnatal rhesus monkey (*Macaca mulatta*) brains were obtained from the California National Primate Research Center. Animals were deeply anesthetized with sodium pentobarbital (50 mg/kg intravenously) and perfused transcardially with ice-cold 1% and 4% paraformaldehyde in 0.1 M phosphate buffer (pH 7.4) according to a standard protocol (6), which conformed to National Institutes of Health guidelines and was approved by the Institutional Animal Care and Use Committee at the University of California at Davis. Brains were postfixed for 6 h in the same fixative, cryoprotected in 10% and 20% glycerol solutions in 0.1 M phosphate buffer (pH 7.4, for 24 and 72 h respectively), rapidly frozen in isopentane and stored at −70 °C until sectioning. Sections were cut at 30 µm on a freezing sliding microtome and stored in cryopreservative at −70 °C prior to immunofluorescent staining in parallel with mouse brain sections.

Mice: Mice were anesthetized with sodium pentobarbital (60 mg/kg) prior to transcardial perfusion with PBS, immediately followed by phosphate-buffered 4% paraformaldehyde, pH 7.3. Perfused brains were postfixed overnight at 4°C in the same fixative before sequential 12 h incubations in 10%, 20%, and 30% sucrose in PBS, pH 7.5. Cryoprotected brains were frozen on dry ice and cut into 40-µm-thick sections with a sliding microtome. Prior to immunofluorescent staining, sections were stored at −20°C in a cryopreservative solution (by volume: 45% PBS, 30% ethylene glycol, 25% glycerol).

Immunofluorescent staining: Brain sections were rinsed several times in PBS before blocking in PBS plus 5% normal goat serum and 0.2% Triton X-100 (NGST) for 1 h at room temperature. Blocked sections were subsequently incubated in primary antibodies diluted in NGST for 48 h at 4°C. Several rinses in PBS containing 0.2% Triton X-100 (PBST) followed, just before

incubation in secondary antibodies (also diluted in NGST) for 1 h at room temperature. In most experiments, 4′,6-diamidino-2-phenylindole (DAPI; Thermo Fisher Scientific, D1306) was also included at a concentration of 700 ng/ml for nuclear counterstaining. Primary antibodies and reagents used included 1:500 mouse anti-NeuN (Millipore, MAB377, RRID:AB_10048713), 1:1,000 mouse anti-UBE3A (Sigma, 1404508, RRID:AB_10740376), rabbit anti-GFAP (DAKO, Z0334), RRID:AB_10013382), 1:1,000 biotinylated WFA (Sigma, L1516, RRID:AB_2620171), 1:1,000 rabbit anti-GABA (Sigma, A2052, RRID:AB_477652), 1:500 rabbit anti-parvalbumin (Swant, PV 25, RRID:AB_10000344), and 1:250 rabbit anti-DARPP-32 (Millipore, AB10518, RRID:AB_10807019). The following secondary antibodies and reagents (Thermo Fisher Scientific) were used at 1:500: Alexa Fluor-647 goat anti-mouse IgG_{2a} (A21241, RRID:AB_141698), Alexa Fluor-488 goat anti-mouse IgG_{2a} (A21131, RRID:AB_141618), Alexa Fluor-647 goat anti-mouse IgG_1 (A21240, RRID:AB_141658), Alexa Fluor-568 goat anti-rabbit IgG (A11036, RRID:AB_143011), and Alexa Fluor-568 streptavidin (S11226, RRID:AB_2315774). All brain sections compared qualitatively and/or quantitatively were stained within the same experiment under identical conditions.

Imaging: Images of immunofluorescently labeled brain sections were acquired with a Zeiss LSM 710 confocal microscope equipped with ZEN imaging Software (Zeiss, RRID:SCR_013672). We collected images for quantitative comparison during the same imaging session using identical acquisition parameters. Quantitative analysis of immunofluorescence intensity and immunolabeled cells was conducted using ImageJ software. Regions of interest corresponding to individual neurons in neocortex and hippocampus (**Fig. 8A and 8E**) were extracted from images of NeuN staining using an automated processing pipeline incorporating Phansalkar thresholding and the watershed separation algorithm.

Supplemental References

1. Zampeta FI, Sonzogni M, Niggl E, Lendemeijer B, Smeenk H, de Vrij FMS, et al. Conserved UBE3A subcellular distribution between human and mice is facilitated by non-homologous isoforms. *Hum Mol Genet.* 2020;29(18):3032-43.
2. Avagliano Trezza R, Punt AM, Mientjes E, van den Berg M, Zampeta FI, de Graaf IJ, et al. Mono-ubiquitination of Rabphilin 3A by UBE3A serves a non-degradative function. *Sci Rep.* 2021;11(1):3007.
3. Bossuyt SNV, Punt AM, de Graaf IJ, van den Burg J, Williams MG, Heussler H, et al. Loss of nuclear UBE3A activity is the predominant cause of Angelman syndrome in individuals carrying UBE3A missense mutations. *Hum Mol Genet.* 2021;30(6):430-42.
4. Schneider CA, Rasband WS, and Eliceiri KW. NIH Image to ImageJ: 25 years of image analysis. *Nat Methods.* 2012;9(7):671-5.
5. Deacon RM. Assessing nest building in mice. *Nat Protoc.* 2006;1(3):1117-9.
6. Lavenex P, Lavenex PB, Bennett JL, and Amaral DG. Postmortem changes in the neuroanatomical characteristics of the primate brain: hippocampal formation. *J Comp Neurol.* 2009;512(1):27-51.

Supplementary Figures and Legends

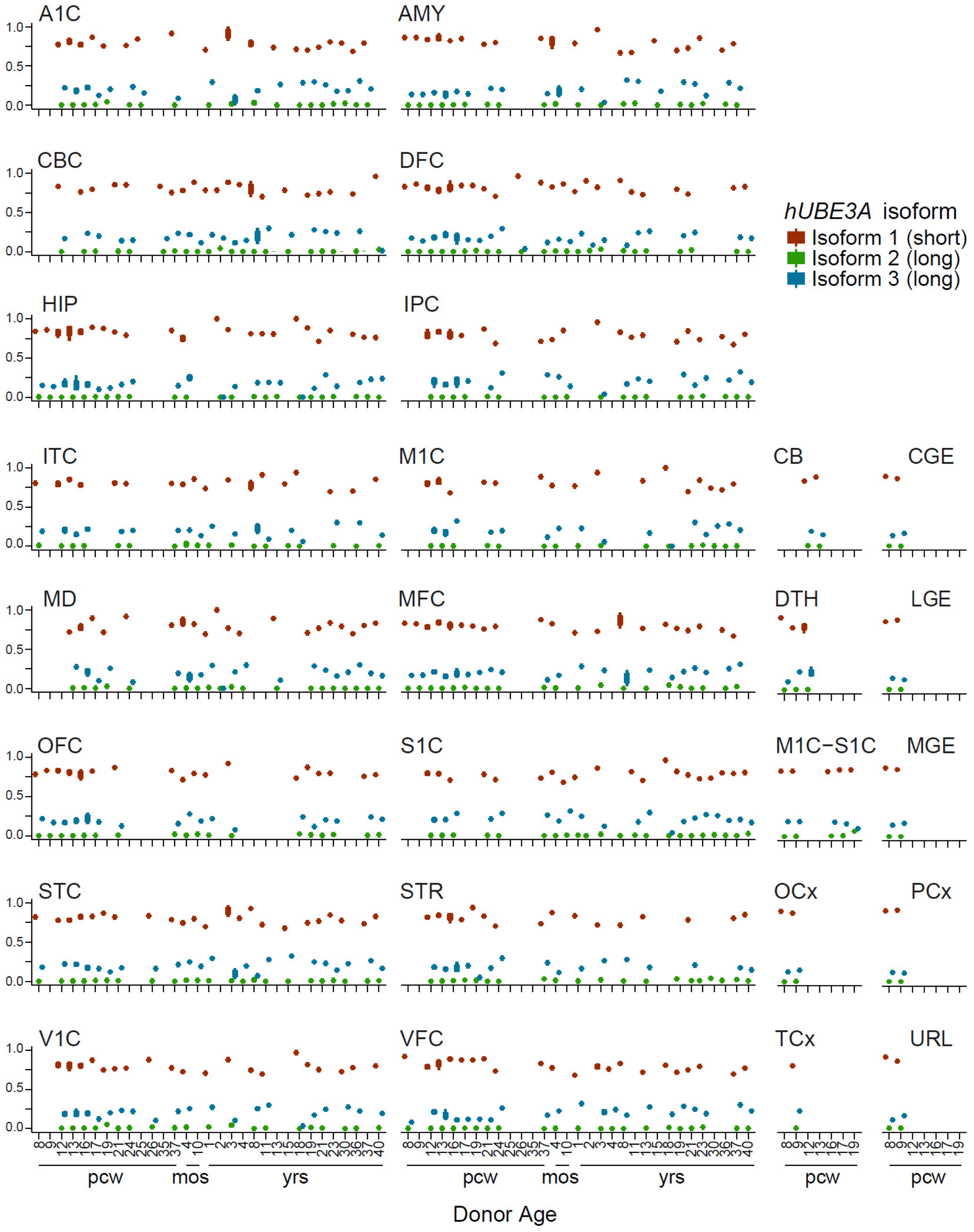

Fig. S1. *UBE3A* isoform expression across human brain regions and development. Boxplots of *hUBE3A* isoform expression in human brain regions across development and aging. Whiskers represent 1.5 x interquartile range. Isoform fractions were estimated from RNA-seq coverage over *hUBE3A* exons including exon 6, which is common to all *hUBE3A* transcripts, and exons 3 and 4, which encode long *hUBE3A* isoforms 3 and 2, respectively, when spliced to exon 6. Exon 3 and 4 reads were weighted according to exon 6 splicing frequencies previously published for human cortex (1). Long *hUBE3A* isoform 3 was computed as (0.322*Exon 3)/Exon 6; long *hUBE3A* isoform 2 = (0.071*Exon 4)/Exon 6; short *hUBE3A* isoform 1 = (Exon 6 – ((0.322*Exon 3) + (0.071*Exon 4)))/Exon 6. Data were plotted for all developmental timepoints and brain regions where available, provided neither exon 3 nor exon 4 values exceeded those of exon 6.

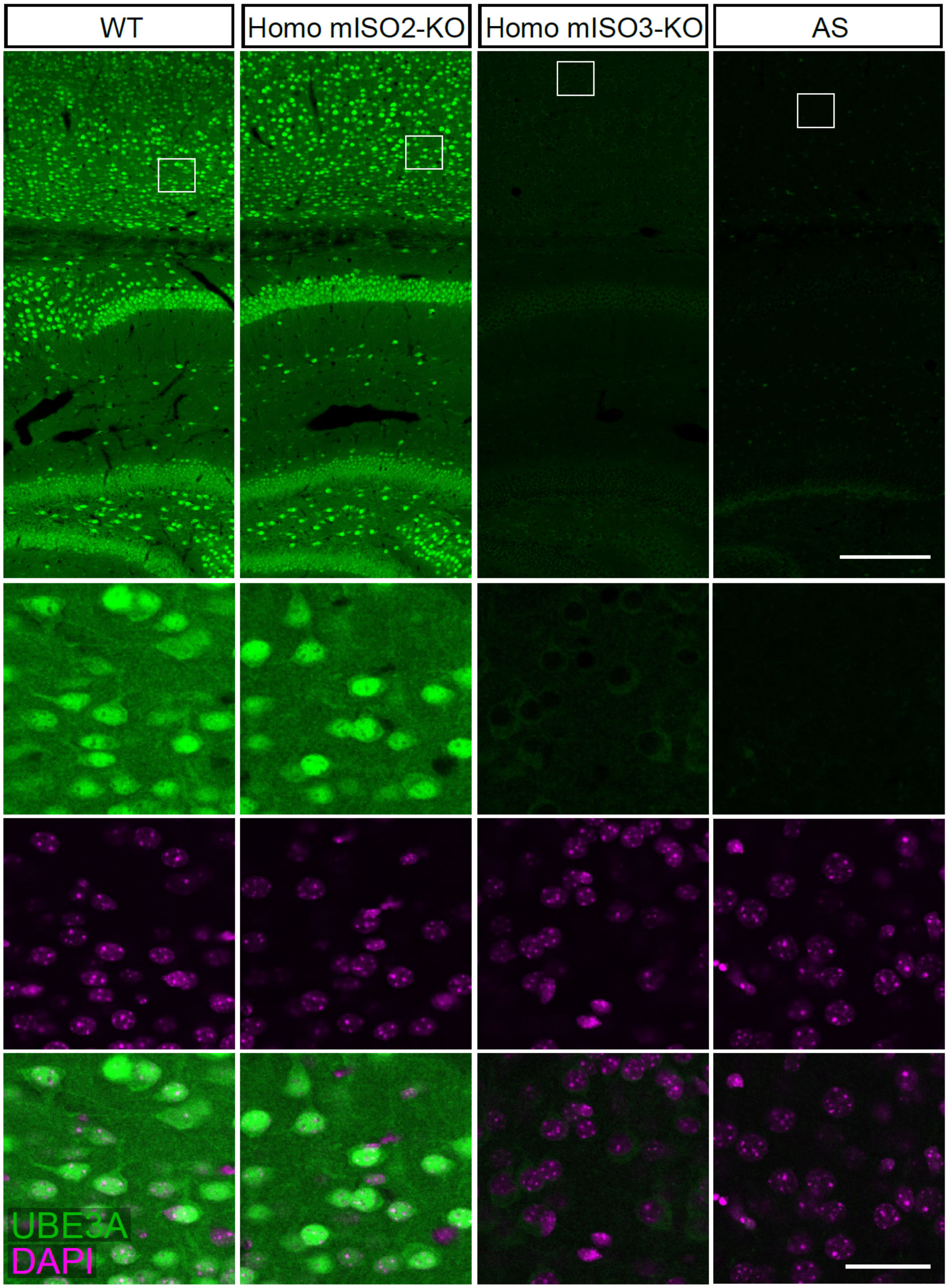

Fig. S2. Subcellular localization of short and long UBE3A isoforms *in vivo*. UBE3A immunofluorescence staining (green) in sections of cortex and hippocampus from P15 WT, homozygous long UBE3A knockout (Homo mISO2-KO), homozygous short UBE3A knockout (Homo mISO3-KO), and AS model mice. Sections are counterstained with DAPI (magenta). Scale bars: 200 µm, 30 µm.

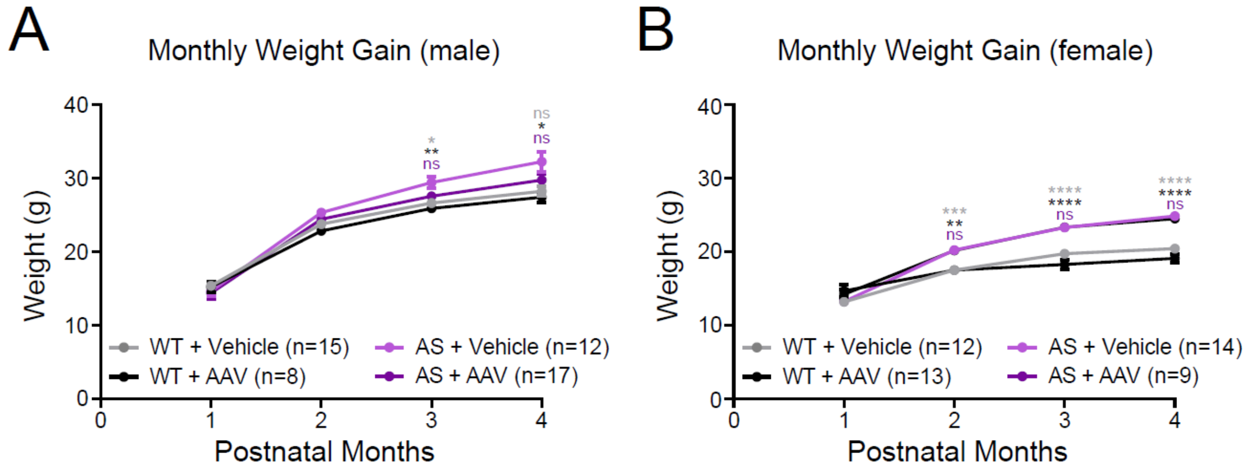

Fig. S3. Neonatal ICV injection of PHP.B/hUBE3Aopt neither affects body growth by one month of age nor mitigates adult-onset obesity in AS model mice. Monthly body weight recorded from male (**A**) and female (**B**) WT and AS model mice following neonatal ICV treatment with 1 µl of either vehicle or 1.6 x 10^{14} vg/ml PHP.B/hUBE3Aopt. All data analyzed by two-way ANOVA with Tukey's *post hoc* testing. Values are means ± SEM. *$P < 0.05$, **$P < 0.01$, ***$P < 0.001$, ****$P < 0.0001$.

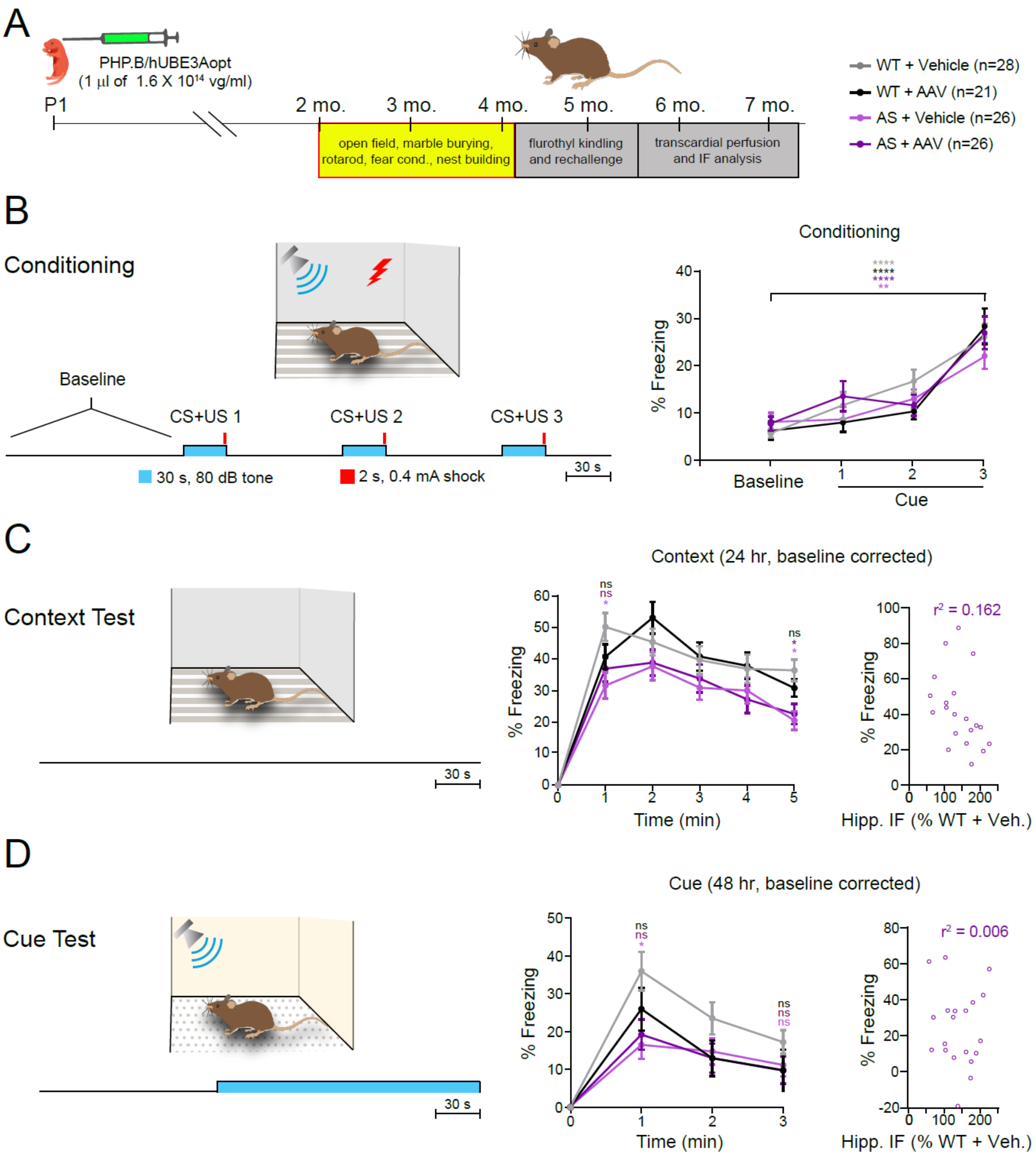

Fig. S4. Fear conditioning analysis in adult AS mice following neonatal ICV injection of PHP.B/hUBE3Aopt vector. (**A**) Experimental timeline for evaluation of behavioral performance in adult AS mice following neonatal ICV administration of 1 µl of 1.6 x 10^{14} vg/ml PHP.B/hUBE3Aopt. Sample sizes for each experimental group are listed to the right. (**B**) Schematic of testing chamber and experimental protocol for fear conditioning by presenting 3 pairings of a conditioned auditory cue (CS, 80 dB tone) and an unconditioned foot shock (US, 0.4 mA). Right panel: quantification of freezing during successive cue presentations. (**C**) Schematic of testing chamber and experimental protocol for testing of contextual fear memory. Middle panel: quantification of freezing in 1 min bins during the 5 min context test. Right panel: correlation analysis (Pearson's r) of normalized hippocampal UBE3A immunofluorescence and contextual freezing (1st minute). (**D**) Schematic of testing chamber and experimental protocol for testing of auditory cue memory. Middle panel: quantification of freezing in 1 min bins during the 3 min tone presentation. Right panel: correlation analysis (Pearson's r) of normalized hippocampal UBE3A immunofluorescence and cued freezing (1st minute). Fear conditioning and memory tasks analyzed by two-way repeated measures ANOVA with Tukey's *post hoc* testing. Values are means ± SEM. **P* < 0.05, ***P* < 0.01, ****P* < 0.001, *****P* < 0.0001.

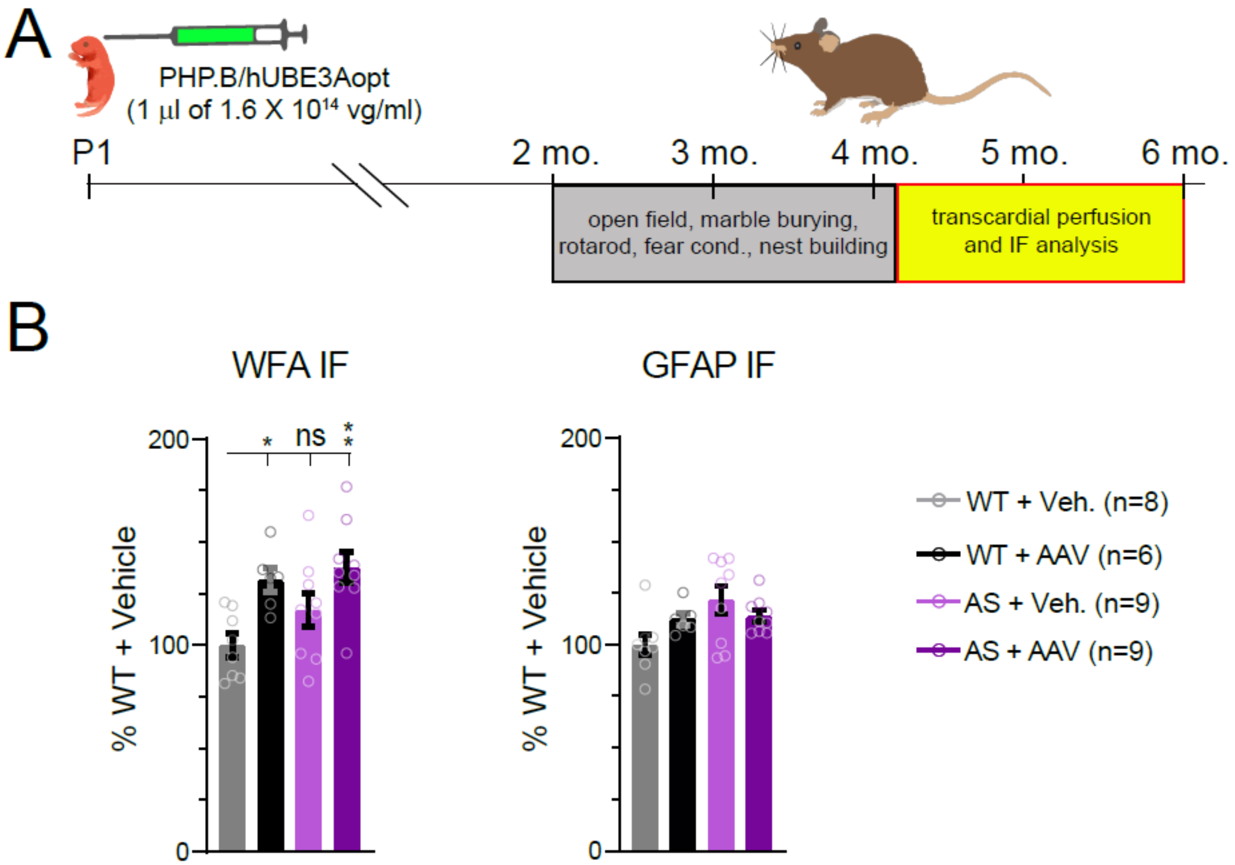

Fig. S5. Analysis of WFA and GFAP immunofluorescence in PHP.B/hUBE3Aopt-treated mice naïve to flurothyl kindling. (**A**) Experimental timeline for immunofluorescence (IF) analysis following behavioral analysis. (**B**) Quantification of normalized mean WFA IF (left panel) and normalized mean GFAP IF (right panel) in the dentate gyrus. One-way ANOVA, Tukey's *post hoc* (WFA); Welch's ANOVA, Dunnett's *post hoc* (GFAP). Values are means ± SEM. *$P < 0.05$, **$P < 0.01$.

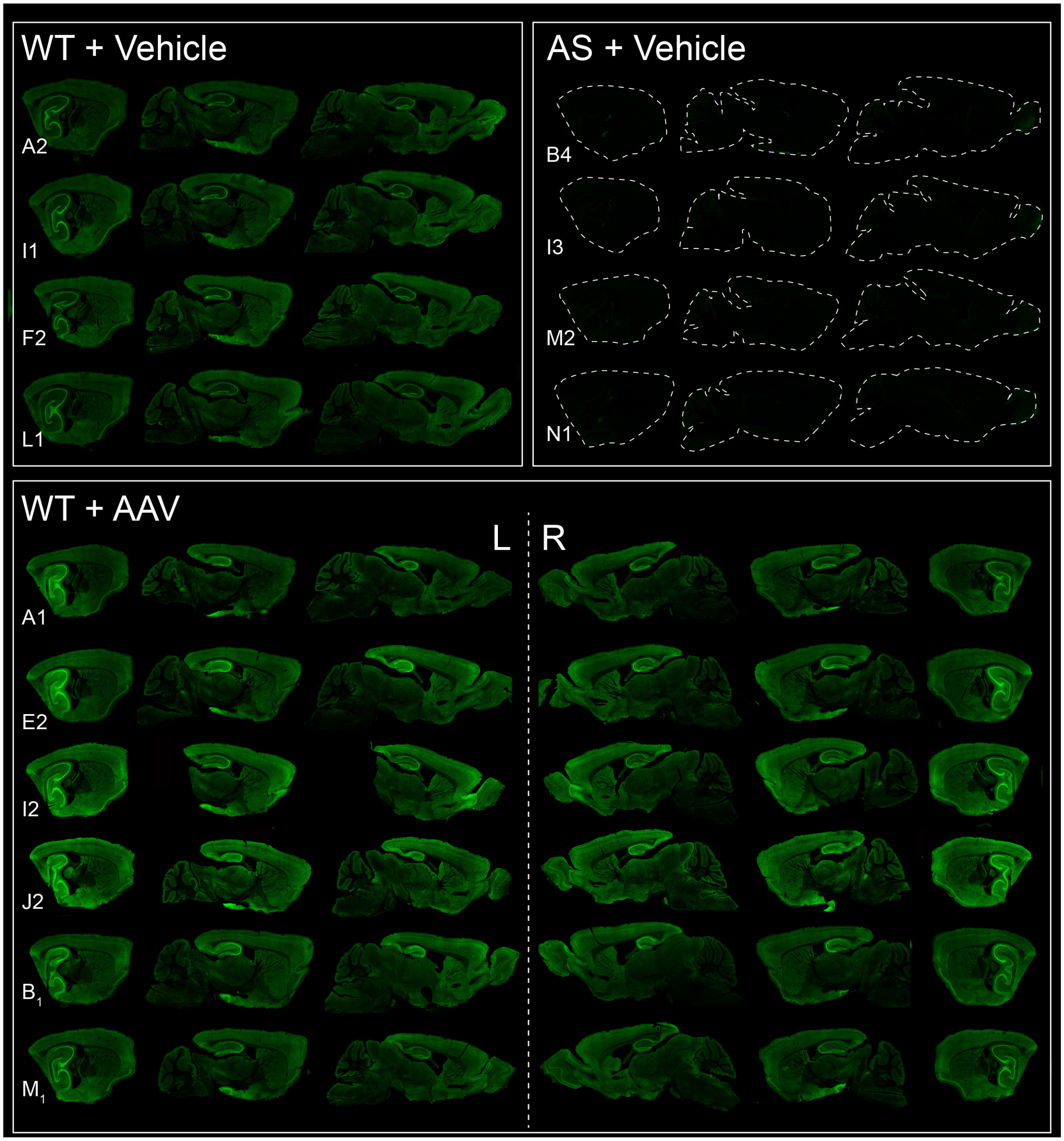
WT + Vehicle
A2
I1
F2
L1
AS + Vehicle
B4
I3
M2
N1
WT + AAV
L
R
A1
E2
I2
J2
B1
M1

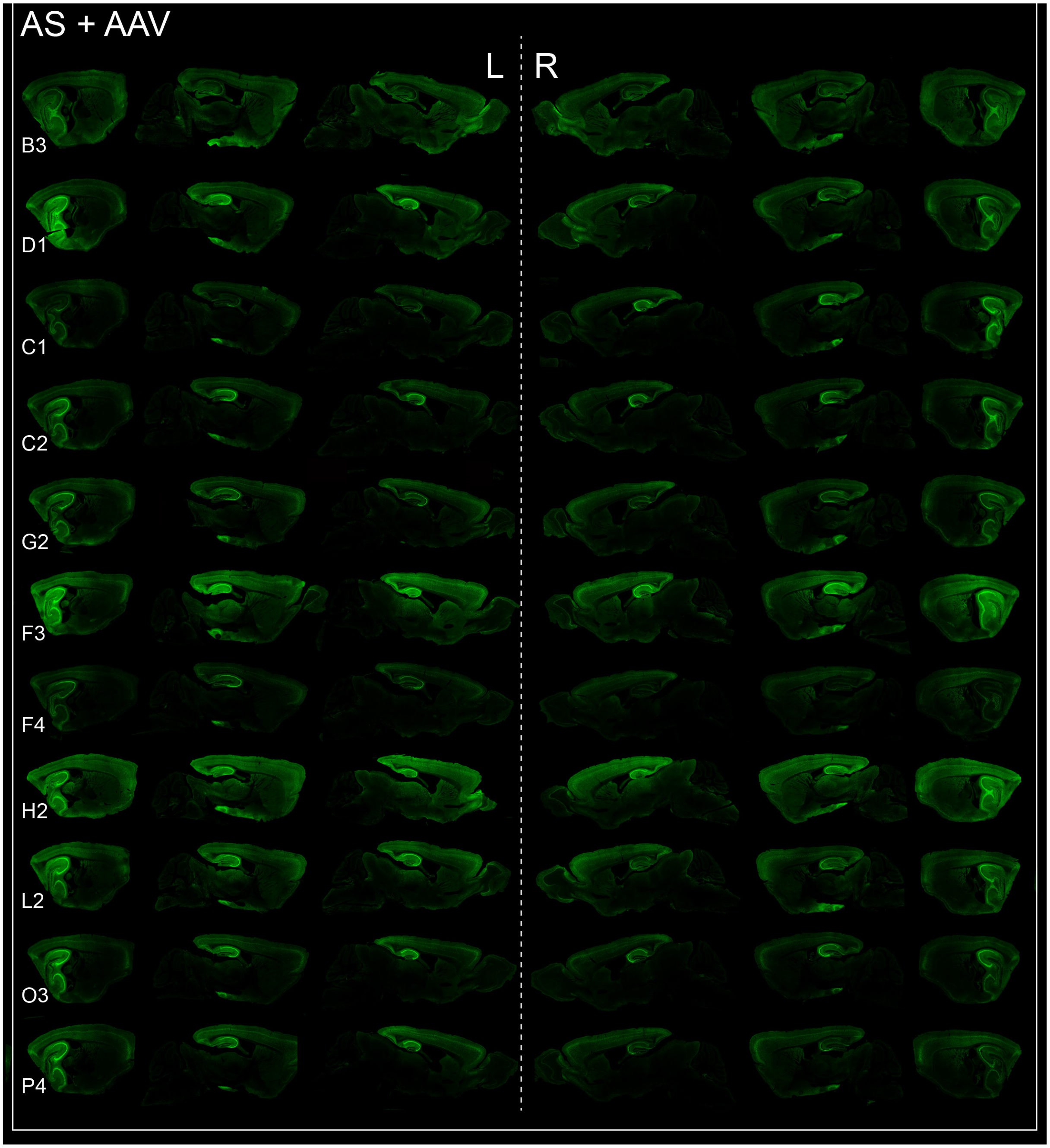

Fig. S6. Compendium of UBE3A immunofluorescence staining in PHP.B/hUBE3Aopt-treated mice. Representative images of UBE3A immunofluorescence in sagittal sections of WT and AS model mice treated intracerebroventricularly with 1 µl of either vehicle or 1.6 x 10^{14} vg/ml PHP.B/hUBE3Aopt as neonates. UBE3A-stained sections from individual animals are organized by row. Sections from left hemispheres (L) are organized in lateral to medial (left to right) arrays. Sections from right hemispheres are ordered in opposing fashion to the right of the dashed line.

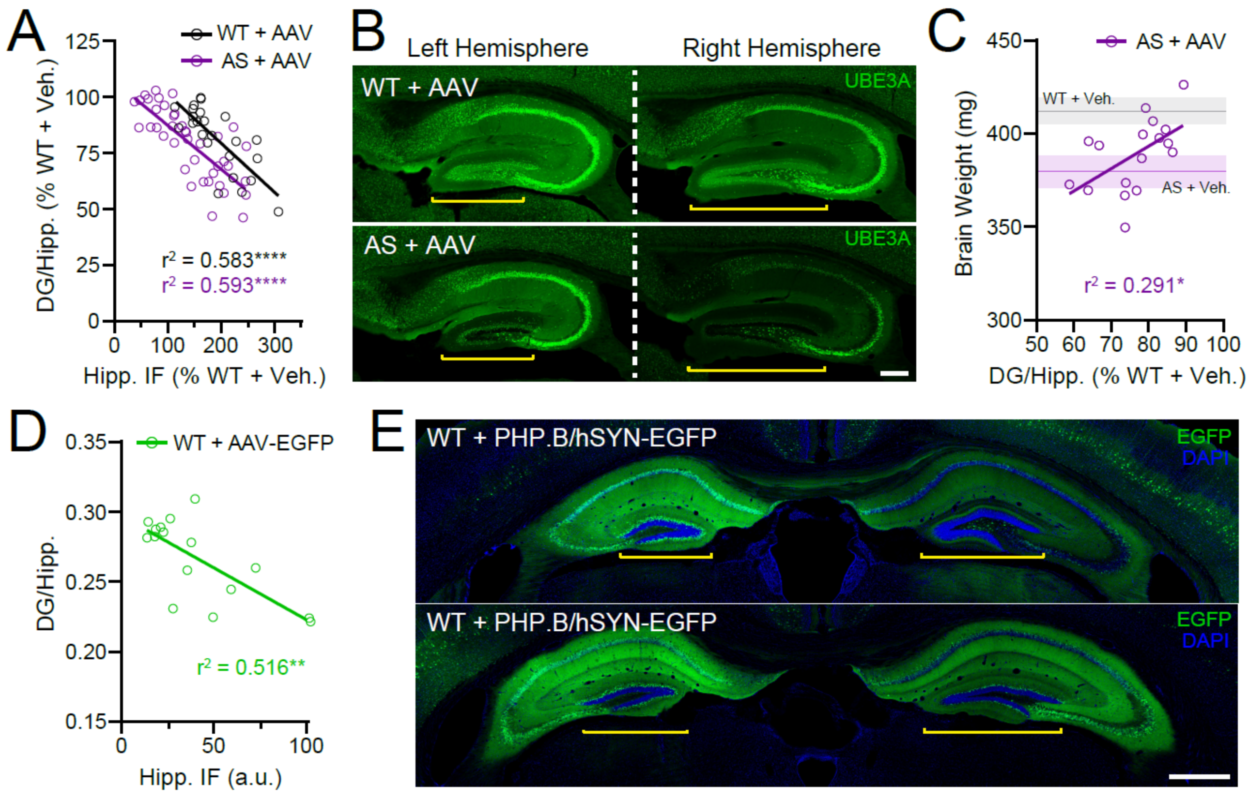

Fig. S7. Analysis of hippocampal pathology due to mistargeting of PHP.B vectors. (**A**) Correlation analysis of normalized hippocampal UBE3A immunofluorescence (IF) and dentate gyrus (DG) volume (expressed as a normalized proportion of the total hippocampus) in WT + AAV and AS + AAV mice. (**B**) Examples of hemispherically biased PHP.B/hUBE3Aopt transduction in WT + AAV and AS + AAV mice. Yellow brackets span each DG. Scale bar, 250 µm. (**C**) Linear regression of proportional DG volume and postmortem brain weight in AS + AAV mice, plotted over the 95% confidence intervals for brain weight for WT + Vehicle (gray band) and AS + Vehicle (light purple band) mice. (**D**) Correlation analysis of normalized hippocampal EGFP IF and proportional DG volume in adult WT mice following neonatal ICV administration of 2 µl of 2.3 x 10^{13} vg/ml PHP.B/hSYN-EGFP. (**E**) Examples of hemispherically biased PHP.B/hSYN-EGFP transduction in WT mice. Yellow brackets span each DG. Scale bar, 500 µm. *$P < 0.05$, **$P < 0.01$, ****$P < 0.0001$.

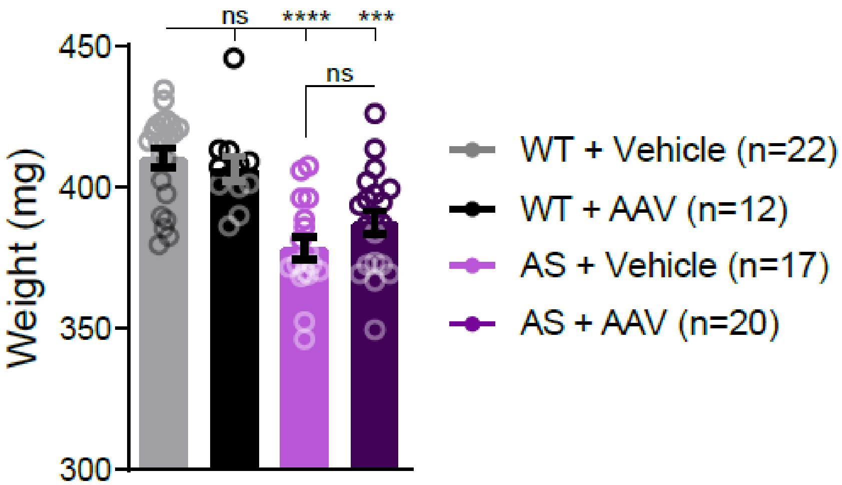

Fig. S8. Postmortem brain weight analysis in adult AS mice following neonatal ICV injection of PHP.B/hUBE3Aopt vector. Mean ± SEM brain weight measured from dissected brains following transcardial perfusion with 4% paraformaldehyde. One-way ANOVA, Tukey's *post hoc*. ***$P < 0.001$, ****$P < 0.0001$.

Full unedited blot for Figure 2C:

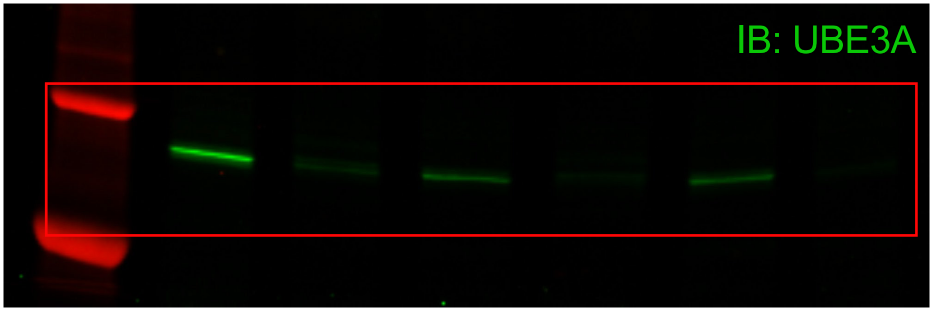

☐ portion shown in Figure 2C.

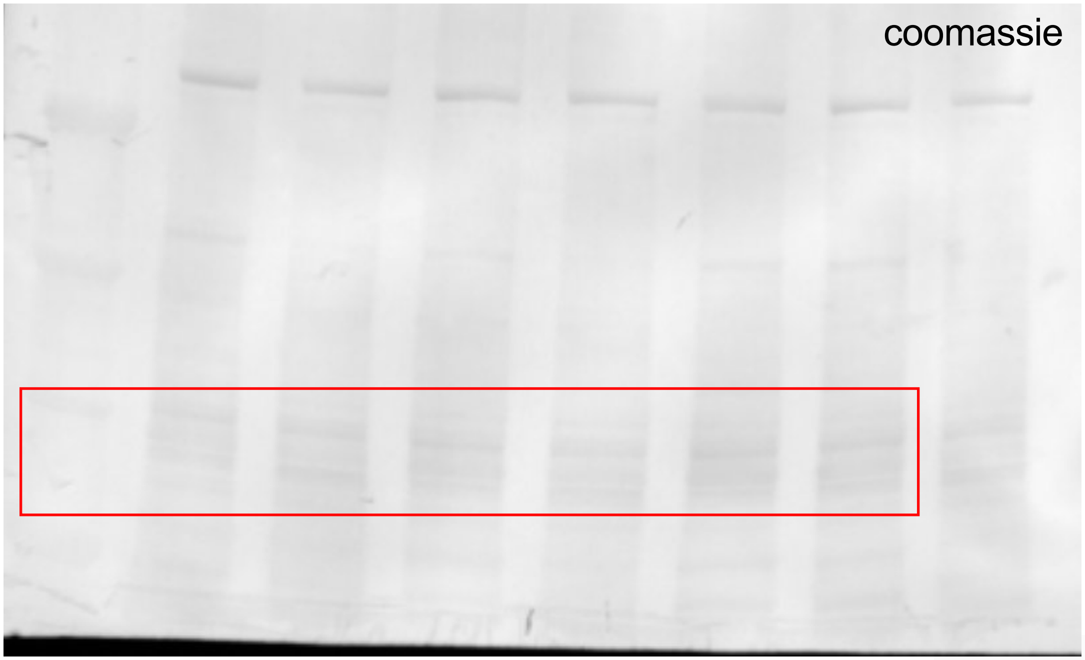

Full unedited blots for Figure 2D:

☐ portions shown in Figure 2D.

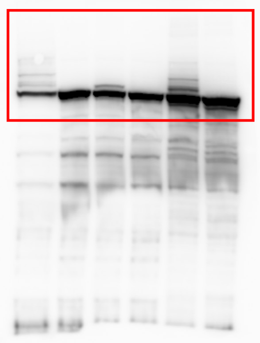

UBE3A auto-ubiquitination (IB: HA)

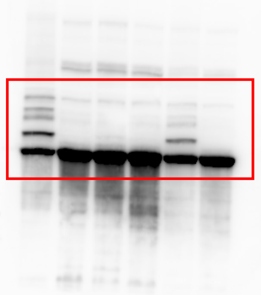

RING1B target ubiquitination (IB: v5)
